# Development and cross-validation of prediction equations for body composition in adult cancer survivors from the Korean National Health and Nutrition Examination Survey (KNHANES)

**DOI:** 10.1371/journal.pone.0309061

**Published:** 2024-10-04

**Authors:** Changwoo Park, Kyuwoong Kim, Minju Kim, Jin Young Choi

**Affiliations:** 1 Linical Korea Co., Ltd, Seoul, Republic of Korea; 2 National Cancer Center, National Cancer Control Institute, Goyang, Republic of Korea; 3 National Cancer Center, Graduate School of Cancer Science and Policy, Goyang, Republic of Korea; University of Mississippi, UNITED STATES OF AMERICA

## Abstract

Epidemiological studies frequently use indices of adiposity related to mortality. However, no studies have validated prediction equations for body composition in adult cancer survivors. We aimed to develop and cross-validate prediction equations for body fat mass (BFM), lean body mass (LBM), trunk fat mass (TFM), and appendicular lean mass (ALM) in adult cancer survivors using sociodemographic, anthropometric, and laboratory test data. This study included adult cancer survivors from the Korean National Health and Nutrition Examination Survey 2008–2011 with complete data on Dual-energy X-ray absorptiometry (DXA) measurements. A total of 310 participants were randomly divided into development and cross-validation groups (5:5 ratio). Age, height, weight, waist circumference, serum creatinine levels, and lifestyle factors were included as independent variables The predictive equations were developed using a multiple linear regression and their predictive performances were primarily evaluated with R^2^ and Concordance Correlation Coefficient (CCC). The initial equations, which included age, height, weight, and waist circumference, showed different predictive abilities based on sex for BFM (total: R^2^ = 0.810, standard error of estimate [SEE] = 3.072 kg, CCC = 0.897; men: R^2^ = 0.848, SEE = 2.217 kg CCC = 0.855; women: R^2^ = 0.791, SEE = 2.194 kg, CCC = 0.840), LBM (total: R^2^ = 0.736, SEE = 3.321 kg, CCC = 0.838; men: R^2^ = 0.703, SEE = 2.450 kg, CCC = 0.774; women: R^2^ = 0.854, SEE = 2.234 kg, CCC = 0.902), TFM (total: *R*^*2*^ = 0.758, SEE = 1.932 kg, CCC = 0.844; men: R^2^ = 0.650, SEE = 1.745 kg, CCC = 0.794; women: R^2^ = 0.852, SEE = 1.504 kg, CCC = 0.890), and ALM (total: R^2^ = 0.775, SEE = 1.726 kg, CCC = 0.876; men: R^2^ = 0.805, SEE = 1.320 kg, CCC = 0.817; women: R^2^ = 0.726, SEE = 1.198 kg, CCC = 0.802). When additional factors, such as creatinine, smoking, alcohol consumption, and physically inactive were included in the initial equations the predictive performance of the equations were generally improved. The prediction equations for body composition derived from this study suggest a potential application in epidemiological investigations on adult cancer survivors.

## Introduction

Body mass index (BMI) serves as a widely employed indicator of adiposity in epidemiological investigations, with documented associations to various adverse health outcomes, including cancer and cardiovascular disease (CVD) [1–8]. Nevertheless, the utility of BMI, derived from weight in kilograms divided by height in meters squared, is constrained by its incomplete representation of body composition, which encompasses both body fat mass (BFM) and lean body mass (LBM). Existing literature has consistently demonstrated a U-shaped association between BMI and all-cause and cause-specific mortality, as well as incident cancer and cardiovascular outcomes [9–12], a phenomenon known as the "obesity paradox [13–19]." This counterintuitive association suggests improved survival outcomes for individuals with overweight or obesity in specific chronic diseases or mortality [20, 21]. Consequently, the application of prediction equations assessing fat mass and lean mass alongside BMI would be imperative in epidemiologic research to ensure a more accurate evaluation of association between body composition and health outcomes. Furthermore, the limited availability of body composition data in population-based cohort studies, possibly owing to high cost and technical requirements for measurements such as Dual X-ray absorptiometry (DXA) or bioelectrical impedance analysis, underscores the need for simple approaches to enhance accessibility in research settings.

Previous studies on development and validation of anthropometric prediction equations for body composition were based on nationally representative samples of non-institutionalized individuals [22, 23]. Two notable prediction equations were derived from the US National Health and Nutrition Examination Survey (NHANES) and the Korea National Health and Nutrition Examination Survey (KNHANES). These equations were used to investigate associations of predicted fat mass and lean mass with CVD and mortality in the US health professionals and health examinees in the Republic of Korea [24, 25]. However, these estimates for body fat mass and lean mass may not be directly applicable to cancer survivors because they are affected by unique changes in body composition, treatment, and lifestyle changes after cancer diagnosis [26, 27]. Cancer treatments, including chemotherapy, radiation, and surgery, often result in significant changes in body composition, including muscle wasting and fat redistribution. Different types of treatments for different cancers can cause drastic changes in body composition, requiring specialized prediction equations for this population. Studies have shown that weight stability can mask significant changes in body composition in cancer patients, such as loss of lean mass despite stable or increased weight [27].

Therefore, our study aimed to develop prediction equations for body fat mass (BFM) and lean body mass (LBM) that are derived from the community-dwelling cancer survivors using data from the KNHANES. Moreover, we also internally validated these estimates to provide more precise predictions of body composition in this specific population.

## Materials and methods

### Study population

**Fig 1:**
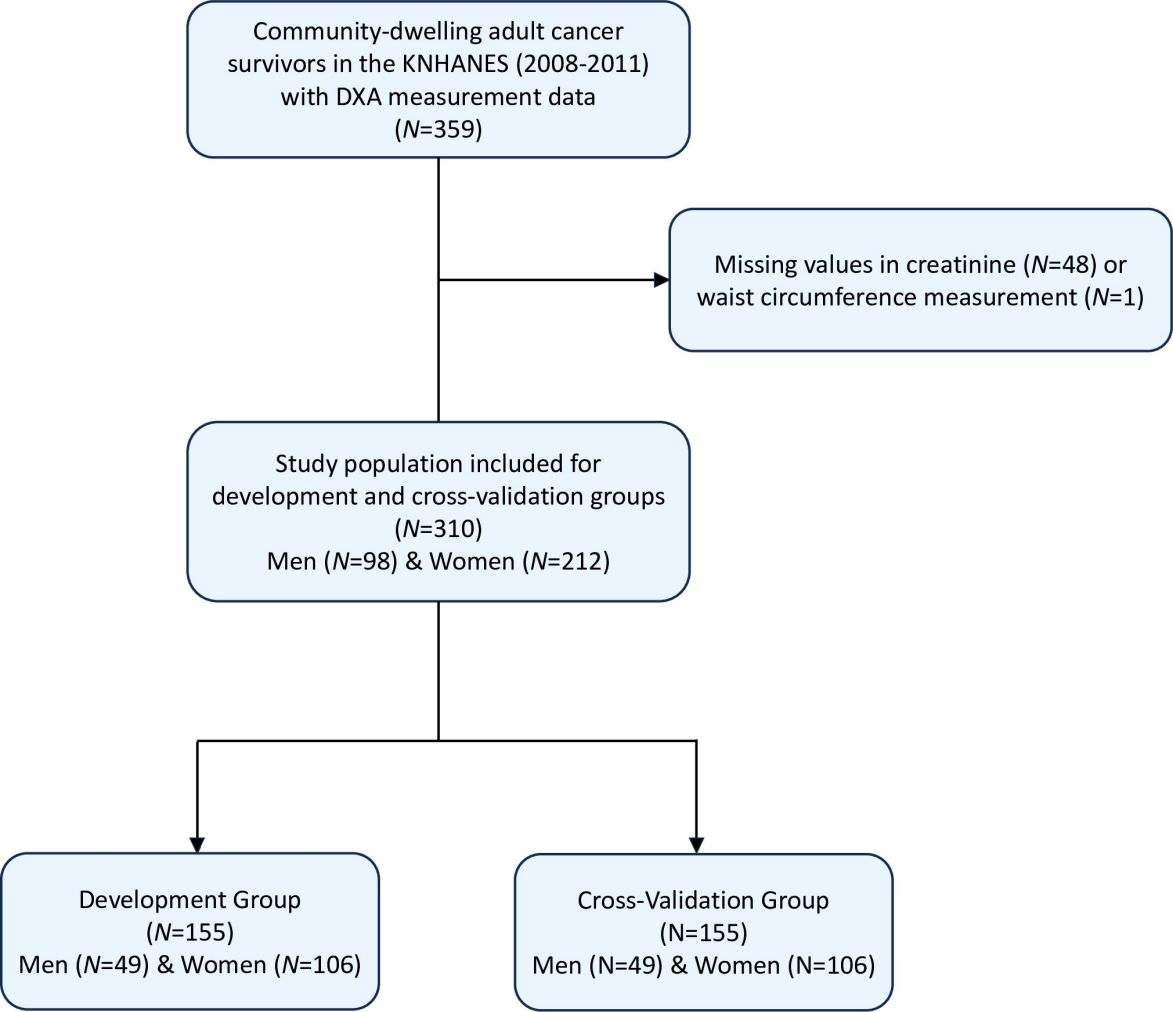
Flow diagram of participant selection from the Korean National Health and Nutrition Examination Survey (2008–2011) for development and cross-validation sets. Acronyms: KNHANES, Korea National Health and Nutrition Examination Survey; DXA, Dual Energy X-ray Absorptiometry.

This study used data on community-dwelling cancer survivors from the fourth and fifth KNHANES (collected from January 1, 2008 to December 31, 2011), a cross-sectional survey carried out by the Korean Disease Control and Prevention Agency (KDCA) to assess health and nutrition status among non-institutionalized civilians sampled through a multistage, probability-cluster survey method in the Republic of Korea. Details of the KNHANES are available in detail elsewhere [28]. The Institutional Review Board (IRB) of the KDCA approved the use of protocols, conduct of research using the KNHANES (IRB No.: 2013-12EXP-03-5C). In adherence to the ethical guidelines, written informed consent was obtained from all participants of the KNHANES before their involvement. All data utilized in the current study underwent full anonymization procedures prior to assessment. We included the individuals with history of cancer diagnosis who received DXA scan from 2008–2011 (n = 359). Information regarding a history of cancer diagnosis is collected through comprehensive interviews and health questionnaires administered to each participant. Specifically, participants were asked about their medical history, including any previous diagnoses of cancer [31]. Among these individuals, those with missing data on serum creatinine (*n* = 48) and waist circumference (*n* = 1) were excluded. The final analysis included data on 310 community-dwelling adult cancer survivors (**Fig 1**). The Institutional Review Board (IRB) of Korea Disease Control and Prevention Agency (KDCA) approved the use of protocols, conduct of research using the Korea National Health and Nutrition Examination Survey (KNHANES) (2013-12EXP-03-5C). These were in compliance with the guidelines of the Declaration of Helsinki. All participants provided informed consent prior to participating in the KNHANES.

### Assessment of key variables

The participants of the KNHANES underwent various assessments. Based on the self-reported questionnaire, which included questions about the frequency and duration of smoking, participants were classified into smokers and nonsmokers, designating current smokers to the former and quitters or never-smokers to the latter category. Furthermore, participants were categorized according to their lifetime alcohol consumption, with a distinction drawn between those with a documented history of lifetime alcohol use and those without. The assessment of participants’ physical activity levels was comprehensive and self-reported, resulting in the classification of individuals into two groups—those consistently engaged in intense physical activity at least once a week and those abstaining from such activities.

### DXA scanner and procedure

The DXA scans performed between 2008 and 2011 utilized specialized DXA machines, specifically the Hologic Discovery model (Hologic, Inc, Bedford, MA, USA) and the GE Lunar Prodigy (GE Healthcare, Madison, WI, USA). These machines were used to measure total body fat mass (BFM), lean body mass (LBM), trunk fat mass (TFM), and appendix lean mass (ALM) at multiple study sites. Measurements were in kilograms and rounded to the nearest tenth. To ensure consistency, target positioning during DXA scans was standardized. Participants were instructed to lie on their backs with their arms at their sides and their legs slightly apart. Technicians were trained to manually adjust the region of interest (ROI) for each scan to ensure accuracy. Annual inspections and calibrations were performed on the DXA machines to prevent calibration drift. This included phantom scanning and cross-calibration checks to maintain the accuracy and reliability of the measurements.

### Serum creatinine measurement

Serum creatinine levels were collected as part of laboratory-based biomarker data in the KNHANES. The analysis was performed using an enzymatic method with a Hitachi Automatic Analyzer 7600 (Hitachi, Tokyo, Japan). Quality control was ensured through regular comparative analysis with reference samples.

### Statistical analysis

The analytical process comprised three distinct phases. Initially, participants underwent random allocation, and were equally divided into a development group and a cross-validation group with a balanced 5:5 distribution (n = 155 for development group and n = 155 for cross-validation group). Test for normal distribution, multicollinearity, and homoscedasticity were conducted with Shapiro-Wilk test, variance inflation factor (VIF), and Breusch-Pegan test, respectively. Comparative analysis of the demographic attributes within the study population entailed t-tests for continuous variables and chi-square tests for categorical variables. Subsequently, the development group underwent the development of prediction equations for body fat mass (BFM), lean body mass (LBM), trunk fat mass (TFM), and appendicular lean mas (ALM) using a multiple linear regression model. This developmental process involved both continuous and categorical variables. Among the continuous variables, age, height (cm), weight (kg), waist circumference (cm), and serum creatinine level (mg/dL) were integrated. Additionally, categorical variables, encompassing smoking status, alcohol consumption, and physical activity, were incorporated. The prediction models were constructed through a stepwise linear regression analysis, utilizing adjusted *R*^2^ (coefficient of determination) and standard error of estimate (SEE) as primary benchmarks to ascertain the optimal model. In addition, we computed and visualized Spearman correlation coefficients for the variables used in the model development. In pursuit of robustness, cross-validation was conducted by juxtaposing predicted values from the development group against those from the cross-validation group.

Comparative evaluation of the fitted models’ R^2^ and SEE values was carried out between the development group and cross-validation groups. We also used boxplots and Bland–Altman plots for visual representation and comparative evaluation to assess the visual agreement between the predicted and measured body composition values. In addition, we used concordance correlation coefficient (CCC) to evaluate the performance of the prediction equations by comparing measured and estimated values of BFM, LBM, TFM, and ALM. To address potential variations associated with obesity status, we stratified the development and cross-validation of the prediction equations by obesity (defined as BMI ≥25.0 kg/m^2^ according to the World Health Organization criteria for Asian population).

Furthermore, we statistically compared the validity of our study’s Equation 1 with predictive equations developed for the general population, Equation 1 from Lee et al. (2021) and Equation 2 from Lee et al. (2018) using the cross-validation group to evaluate accuracy of estimating body composition in cancer survivors.

All statistical analyses were performance with SAS software version 9.4 (SAS Institute, Cary, NC, USA) and R software version 3.3.2. Statistical significance was set to *P*<0.05 for all comparisons.

## Results

### Participant characteristics

**Fig 2:**
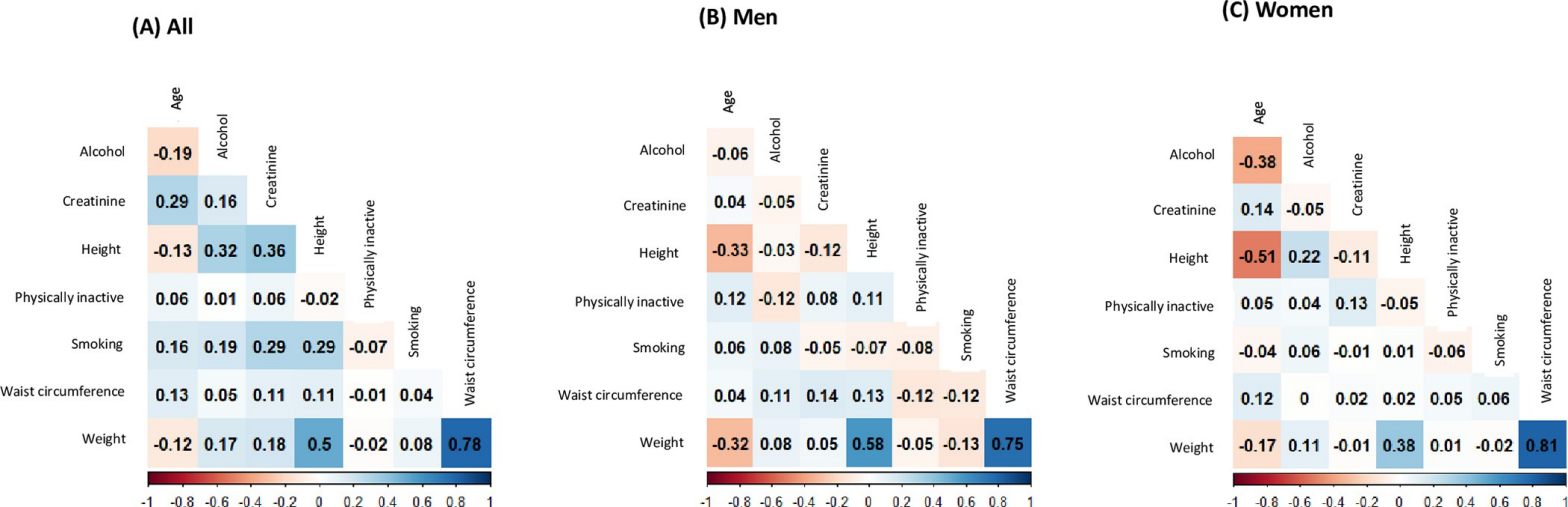
Spearman correlation coefficients for variables used for prediction of body fat mass and lean body mass in adult cancer survivors in the Korea National Health and Nutrition Examination Survey (2008–2011).

The inclusion of participants and derivation of development and cross-validation group are demonstrated in Fig 1. Shapiro-Wilk test, VIF, and Breusch-Pegan test showed evidence for normal distribution, low multicollinearity (VIF<20), and constant variability across the variables. Spearman correlation coefficients between variables used in the model development are presented in Fig 2. Table 1 presents the characteristics of the participants stratified by sex in the development and cross-validation groups. The mean age was 59.6 years (standard deviation [SD] = 11.9 years) for the overall population, 64.6 years (SD = 10.5 years) for males, and 57.3 years (SD = 11.8 years) for females. In the development group, women had a higher mean total LBM (18.9 kg, SD = 6.1) than men (12.2 kg, SD = 4.5), and men had a higher mean total BFM (44.4 kg, SD = 5.7) than women (34.0 kg, SD = 4.8). Men also had a higher ALM (20.9 kg, SD = 3.0) than women (15.2 kg, SD = 2.3). In contrast, women had higher TFM (10.4 kg, SD = 3.9) than men (7.1 kg, SD = 3.0). Most participants reported consuming alcohol (approximately 78.1%). Of the types of cancer included in the study, breast cancer was the most common, followed by stomach cancer, and cervix uteri cancer. Overall, no significant differences were observed in body composition, smoking status, alcohol consumption, physical activity or type of cancer between the development and cross-validation groups.

**Table 1:** Characteristics of community-dwelling adult cancer survivors in the development group and cross-validation group from the Korea National Health and Nutrition Examination Survey (2008–2011).

Variables	Development group						Cross-validation group								
	All (n = 155)		Men (n = 49)		Women (n = 106)		All (n = 155)		Men (n = 51)		Women (n = 104)		*P*_*All*_	*P*_*Men*_	*P*_*Women*_
	Mean (SD)	N (%)	Mean (SD)	N (%)	Mean (SD)	N (%)	Mean (SD)	N (%)	Mean (SD)	N (%)	Mean (SD)	N (%)			
**Age (years)**	59.6 (11.9)		64.6 (10.5)		57.3 (11.8)		60.2 (11.7)		65.6 (9.4)		57.5 (11.9)		0.7	0.6	0.9
**Height (cm)**	158.6 (7.9)		165.8 (7.4)		155.2 (5.5)		159.1 (8.3)		167.6 (6.1)		155.0 (5.7)		0.5	0.2	0.7
**Weight (kg)**	59.3 (10.0)		62.4 (9.0)		57.9 (10.2)		59.7 (9.7)		64.1 (9.3)		57.5 (9.1)		0.8	0.4	0.8
**Waist circumference (cm)**	81.0 (9.4)		82.1 (8.7)		80.5 (9.7)		80.9 (9.7)		83.0 (9.1)		79.9 (9.8)		0.9	0.6	0.6
**Creatinine (mg/dL)**	0.8 (0.3)		1.0 (0.4)		0.7 (0.1)		0.8 (0.2)		1.0 (0.2)		0.7 (0.1)		0.9	0.4	0.5
**Total lean mass (kg)**	16.8 (6.5)		12.2 (4.5)		18.9 (6.1)		16.9 (6.1)		13.3 (5.1)		18.7 (5.8)		0.9	0.3	0.8
**Total Fat mass (kg)**	37.3 (7.0)		44.4 (5.7)		34.0 (4.8)		37.5 (7.3)		45.0 (6.5)		33.8 (4.3)		0.8	0.6	0.8
**Appendicular lean mass (kg)**	17.0 (3.6)		20.9 (3.0)		15.2 (2.3)		17.1 (3.9)		21.2 (3.3)		15.0 (2.1)		0.9	0.6	0.5
**Trunk Fat mass (kg)**	9.3 (3.9)		7.1 (2.9)		10.4 (3.9)		9.5 (3.8)		7.7 (3.4)		10.4 (3.7)		0.7	0.3	1.0
Smoking status (%)^a^	18.7		49.0		4.7		17.4		41.2		5.8		0.8	0.4	0.7
Alcohol consumption (%)^a^	78.1		95.9		69.8		78.1		96.1		69.2		1.0	1.0	0.9
Physically active (%)^a^	73.5		69.4		75.5		74.8		76.5		74.0		0.8	0.4	0.8
Type^b^													0.1	0.2	0.2
** Liver**		9 (5.8)		7 (14.3)		2 (1.9)		5 (3.2)		4 (7.8)		1 (1.0)			
** Thyroid**		18 (11.6)		2 (4.1)		16 (15.1)		14 (9.0)		1 (2.0)		13 (12.5)			
** Colon**		21 (13.5)		13 (26.5)		8 (7.5)		16 (10.3)		7 (13.7)		9 (8.7)			
** Stomach**		33 (21.3)		23 (46.9)		10 (9.4)		53 (34.2)		33 (64.7)		20 (19.2)			
** Breast**		38 (24.5)				38 (35.8)		26 (16.8)				26 (25.0)			
** Cervix uteri**		31 (20.0)				31 (29.2)		30 (19.4)				30 (28.8)			
** Corpus uteri**								2 (1.3)				2 (1.9)			
** Prostate**		1 (0.6)		1 (2.0)											
** Rectum**		1 (0.6)		1 (2.0)											
** Lung**		3 (1.9)		2 (4.1)		1 (0.9)		9 (5.8)		6 (11.8)		3 (2.9)			

NOTE: Prediction group and validation group were divided into 50:50 ratio. P-value from paired t-test for continuous variables and chi-squared test for categorical variables. The study participants included community-dwelling adult cancer survivors with dual-energy X-ray absorptiometry (lean mass and fat mass), anthropometric measurement (height, weight, waist circumference), and laboratory data (creatinine).

^a^Defined as a binary variable (yes/no)

^b^Categorized by the type of cancer diagnosis

Acronym: SEE, standard error of estimate

### Prediction equations

**Fig 3:**
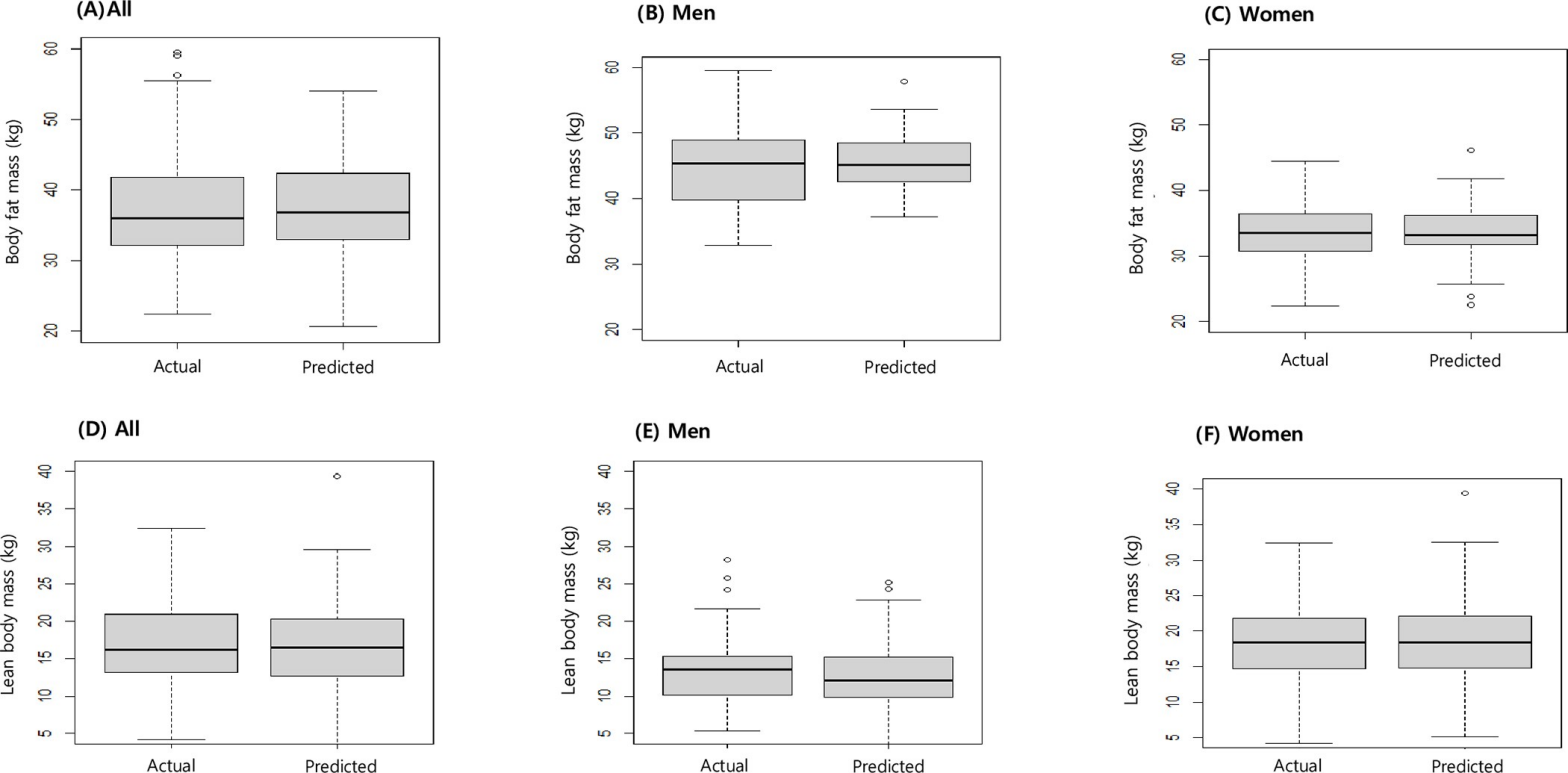
Boxplots comparing actual and predicted values of body fat mass and lean body mass in adult cancer survivors in the Korean National Health and Nutrition Examination Survey (2008–2011).

**Fig 4:**
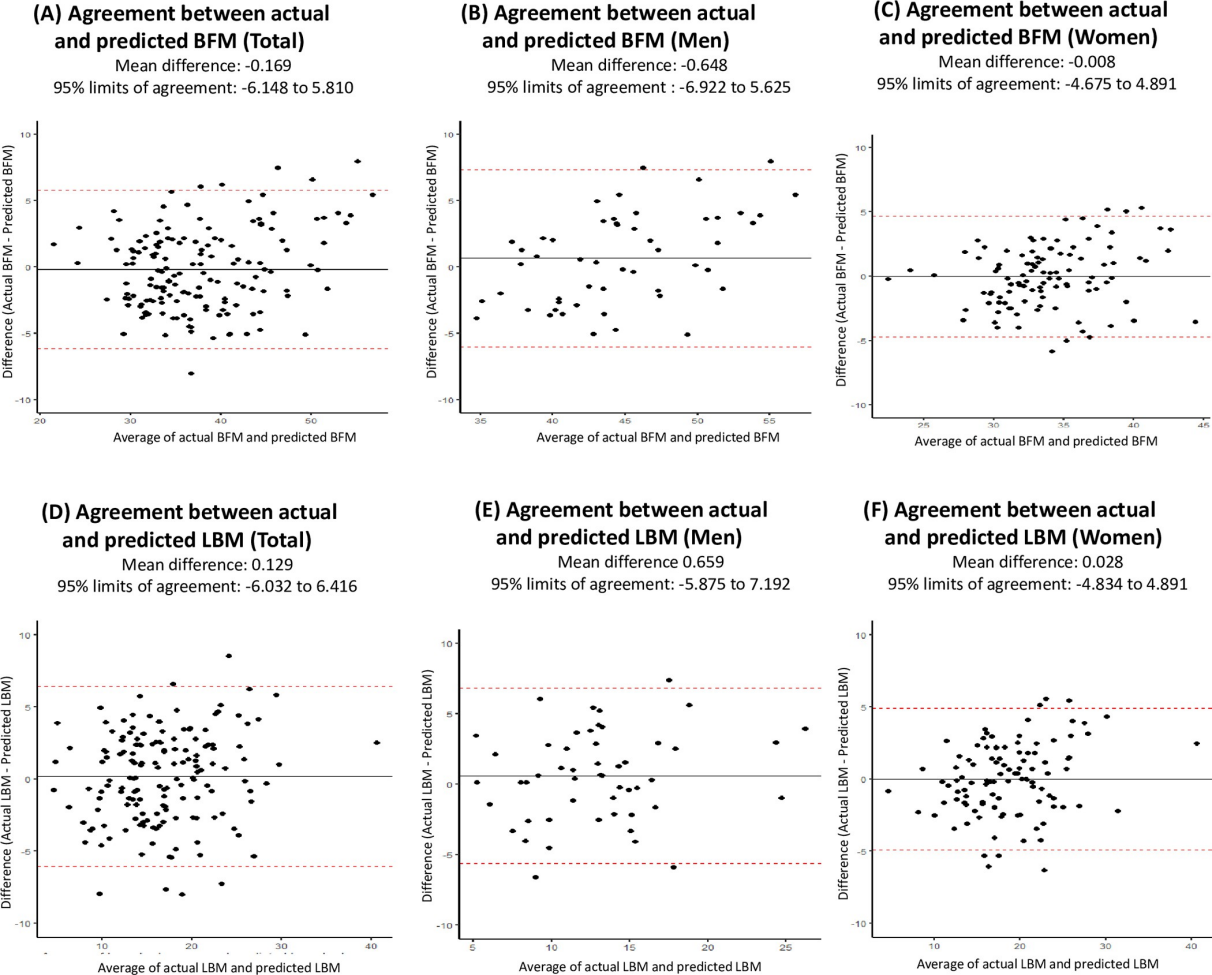
Bland–Altman plots for evaluating differences in actual and predicted values of body fat mass (BFM) and lean body mass (LBM) in adult cancer survivors in the Korean National Health and Nutrition Examination Survey (2008–2011). Acronyms: BFM, body fat mass; LBM, lean body mass.

A visual comparison of DXA measurement and predicted values of BFM and LBM is presented as boxplots in Fig 3. Bland–Altman plots showing agreement between measured and predicted values of BFM and LBM are presented in Fig 4.

### Development and cross-validation of equations to predict body fat mass

Table 2 outlines the formulated equations for predicting BFM. The foundational model, incorporating age, height, weight, and waist circumference, elucidated 81.0% of the BFM variance (R^2^ = 0.81). Expanding the model to include serum creatinine, smoking, and alcohol consumption improved the R^2^ from 0.810 to 0.843, coupled with a 9.05% reduction in SEE (i.e., from 3.072 to 2.794), indicative of enhanced predictive accuracy. The incorporation of physically inactive individuals did not yield a statistically significant enhancement in the model’s R^2^ value. Among men, the basic model demonstrated the highest model fitness (R^2^ = 0.848), while in women, a slight improvement was noted with the inclusion of serum creatinine. Subsequent evaluation in the cross-validation group revealed no significant disparities between measured and predicted weights using DXA in either men or women (**S1 Table**).

**Table 2:** Anthropometric prediction equations for body fat mass in the community-dwelling cancer survivors derived the Korea National Health and Nutrition Examination Survey (2008–2011).

Body fat mass	Intercept	Age (years)	Height (cm)	Weight (kg)	Waist circumference (cm)	Creatinine (mg/dL)	Smoking	Alcohol consumption	Physically inactive	*R* ^2^	SEE
**Total(n = 155)**											
**Equation 1**	-58.300	0.125	0.435	0.449	-0.092*					0.810	3.072
**Equation 2**	-54.438	0.106	0.404	0.472	-0.119*	3.695				0.829	2.917
**Equation 3**	-49.115	0.095	0.375	0.491	-0.130*	2.903	2.155			0.840	2.823
**Equation 4**	-48.415	0.109	0.363	0.488	-0.133*	2.713	1.914	1.232		0.843	2.794
**Equation 5**	-48.436	0.109	0.363	0.488	-0.133*	2.708	1.931	1.225	0.073	0.842	2.804
**Men(n = 49)**											
**Equation 1**	-8.265	0.052	0.154	0.611	-0.174*					0.848	2.217
**Equation 2**	-6.771	0.051	0.145	0.594	-0.151*	-0.773*				0.847	2.225
**Equation 3**	-7.315	0.049	0.145	0.606	-0.156*	-0.826*	0.681			0.847	2.224
**Equation 4**	-8.534	0.055	0.148	0.611	-0.168*	-0.677*	0.587	0.951		0.845	2.243
**Equation 5**	-5.767	0.040	0.128	0.610	-0.158*	-0.905*	0.883	0.942	1.308	0.853	2.186
**Women(n = 106)**											
**Equation 1**	-6.073	0.023	0.115	0.440	-0.058*					0.791	2.194
**Equation 2**	-12.006	0.016	0.137	0.415	-0.041*	4.276				0.800	2.146
**Equation 3**	-11.518	0.016	0.133	0.412	-0.037*	4.216	-0.743*			0.799	2.151
**Equation 4**	-11.831	0.020	0.133	0.410	-0.037*	4.191	-0.761*	0.243		0.798	2.160
**Equation 5**	-11.805	0.020	0.133	0.410	-0.037*	4.165	-0.761*	0.237	0.044	0.795	2.171

*Denotes statistical significance (*P*<0.05)

Acronym: SEE, standard error of estimate

### Development and cross-validation of equations to predict lean body mass

Table 3 shows the proposed equations for the prediction of LBM. The simplest model included age, height, weight, and waist circumference and explained 73.6% of the variance in LBM (R^2^ = 0.736). When serum creatinine levels and lifestyle factors (smoking status and alcohol consumption) were included in the model for the overall population, the *R*^2^ increased from 0.736 to 0.788, and SEE decreased by 11.7% (i.e., from 3.321 to 2.974), indicating an improvement in the predictive ability of the model. For men, including serum creatinine, smoking status, alcohol consumption, and physical activity in the model increased the *R*^2^ from 0.703 to 0.732, and the SEE decreased 5.2% (i.e., from 2.450 to 2.329), indicating the improved predictive ability of the model. For women, predictive ability slightly improved with the inclusion of serum creatinine levels. When the equations were further evaluated in the cross-validation group, no significant differences were found between the measured and predicted weights using DXA in either men or women (**S2 Table**).

**Table 3:** Anthropometric prediction equations for lean body mass in the community-dwelling cancer survivors derived from the Korea National Health and Nutrition Examination Survey (2008–2011).

Lean body mass	Intercept	Age (years)	Height (cm)	Weight (kg)	Waist circumference (cm)	Creatinine (mg/dL)	Smoking	Alcohol consumption	Physically inactive	*R* ^2^	SEE
**Total(n = 155)**											
**Equation 1**	60.736	-0.125*	-0.471*	0.537	0.077					0.736	3.321
**Equation 2**	56.340	-0.103*	-0.436*	0.511	0.109	-4.206*				0.765	3.133
**Equation 3**	49.707	-0.089*	-0.400*	0.488	0.123	-3.219*	-2.685*			0.785	2.992
**Equation 4**	49.082	-0.102*	-0.389*	0.490	0.125	-3.050*	-2.470*	-1.100*		0.788	2.974
**Equation 5**	49.131	-0.101*	-0.389*	0.491	0.125	-3.037*	-2.511*	-1.085*	-0.176*	0.787	2.983
**Men(n = 49)**											
**Equation 1**	4.687	-0.046*	-0.150*	0.363	0.154					0.703	2.450
**Equation 2**	3.601	-0.045*	-0.144*	0.375	0.138	0.562				0.698	2.470
**Equation 3**	4.659	-0.043*	-0.144*	0.353	0.147	0.664	-1.324*			0.715	2.403
**Equation 4**	5.998	-0.049*	-0.147*	0.347	0.160	0.500	-1.221*	-1.045*		0.710	2.423
**Equation 5**	2.533	-0.030*	-0.122*	0.348	0.147	0.786	-1.592*	-1.033*	-1.638*	0.732	2.329
**Women(n = 106)**											
**Equation 1**	5.182	-0.015*	-0.133*	0.544	0.047					0.854	2.334
**Equation 2**	10.846	-0.008*	-0.155*	0.567	0.032	-4.082*				0.859	2.296
**Equation 3**	10.357	-0.008*	-0.151*	0.571	0.027	-4.022*	0.745			0.858	2.302
**Equation 4**	10.366	-0.009*	-0.151*	0.571	0.027	-4.021*	0.745	-0.008*		0.857	2.314
**Equation 5**	10.276	-0.007*	-0.150*	0.571	0.027	-3.931*	0.744	0.014*	-0.154*	0.856	2.325

*Denotes statistical significance (*P*<0.05)

Acronym: SEE, standard error of estimate

### Development and cross-validation of equations to predict trunk fat mass

Table 4 presents the proposed equations for the prediction of TFM. The simplest model, including age, height, weight, and waist circumference, explained 75.8% of the variance in TFM (R^2^ = 0.758). When serum creatinine and smoking status were included, the *R*^2^ increased from 0.758 to 0.786, and SEE decreased from 1.932 kg to 1.816 kg, indicating an improved predictive ability. For men, the inclusion of serum creatinine concentration, smoking status, and physical activity increased the *R*^2^ from 65.0% to 67.1%, and SEE decreased from 1.745 kg to 1.693 kg, indicating a slight improvement in predictive ability. The simplest model showed the highest predictive ability for women. No significant differences were found between the measured and predicted values using DXA in either men or women when the equations were further evaluated in the cross- validation group (**S3 Table**).

**Table 4:** Anthropometric prediction equations for trunk fat body mass in the community-dwelling cancer survivors derived the Korea National Health and Nutrition Examination Survey (2008–2011).

Trunk fat mass	Intercept	Age (years)	Height (cm)	Weight (kg)	Waist circumference (cm)	Creatinine (mg/dL)	Smoking	Alcohol consumption	Physically inactive	*R* ^2^	SEE
**Total (n = 155)**											
**Equation 1**	26.929	-0.051*	-0.256*	0.281	0.115					0.758	1.932
**Equation 2**	24.960	-0.041*	-0.240*	0.269	0.129	-1.884*				0.773	1.871
**Equation 3**	21.698	-0.034*	-0.222*	0.258	0.136	-1.398*	-1.321*			0.786	1.816
**Equation 4**	21.464	-0.039*	-0.218*	0.259	0.137	-1.335*	-1.240*	-0.413*		0.786	1.816
**Equation 5**	21.557	-0.037*	-0.219*	0.260	0.136	-1.309*	-1.318*	-0.384*	-0.337*	0.786	1.816
**Men (n = 49)**											
**Equation 1**	1.619	-0.013*	-0.101*	0.208	0.122					0.650	1.745
**Equation 2**	0.660	-0.012*	-0.095*	0.219	0.108	0.496				0.646	1.756
**Equation 3**	1.335	-0.011*	-0.095*	0.205	0.113	0.562	-0.844*			0.659	1.721
**Equation 4**	1.600	-0.012*	-0.096*	0.204	0.116	0.529	-0.824*	-0.207*		0.651	1.742
**Equation 5**	-0.615	0.000*	-0.080*	0.205	0.107	0.712	-1.061*	-0.199*	-1.047*	0.671	1.693
**Women (n = 106)**											
**Equation 1**	-1.212	0.003	-0.089*	0.275	0.116					0.852	1.504
**Equation 2**	-0.011	0.005	-0.094*	0.280	0.113	-0.866*				0.851	1.509
**Equation 3**	-0.547	0.004	-0.089*	0.283	0.107	-0.800*	0.817			0.851	1.506
**Equation 4**	-0.779	0.007	-0.089*	0.282	0.107	-0.819*	0.804	0.180		0.850	1.512
**Equation 5**	-1.047	0.011	-0.089*	0.283	0.108	-0.551*	0.799	0.243	-0.456*	0.851	1.507

*Denotes statistical significance (*P*<0.05)

Acronym: SEE, standard error of estimate

### Development and cross-validation of equations to predict appendicular lean body mass

Table 5 delineates the proposed equations for the prediction of ALM. The simplest model, including age, height, weight, and waist circumference, explained 77.5% of the variance in ALM (R^2^ = 0.775). When serum creatinine, smoking status, and alcohol consumption were included in the model for the overall population, the *R*^2^ increased from 0.775 to 0.809, and SEE decreased by 8.6% (i.e., from 1.726 to 1.589), indicating an improved predictive ability. For men, including serum creatinine concentration, smoking status, alcohol consumption, and physical activity in the model increased the *R*^2^ from 0.805 to 0.811, and SEE decreased by 1.5% (i.e., from 1.320 to 1.301), indicating a slight improvement in predictive ability. For women, the model showed a slight improvement in predictive ability when the serum creatinine concentration was included. When the equations were further evaluated in the cross-validation group, no significant differences were found between the measured and predicted weights using DXA in either men or women (**S4 Table**).

**Table 5:** Anthropometric prediction equations for appendicular lean body mass in the community-dwelling cancer survivors derived the Korea National Health and Nutrition Examination Survey (2008–2011).

Appendicular lean mass	Intercept	Age (years)	Height (cm)	Weight (kg)	Waist circumference (cm)	Creatinine (mg/dL)	Smoking	Alcohol consumption	Physically inactive	*R* ^2^	SEE
**Total (n = 155)**											
**Equation 1**	-33.676	0.059	0.249	0.210	-0.059*					0.775	1.726
**Equation 2**	-31.602	0.049	0.233	0.222	-0.074*	1.985				0.795	1.647
**Equation 3**	-29.173	0.044	0.219	0.231	-0.079*	1.624	0.984			0.803	1.614
**Equation 4**	-28.698	0.054	0.211	0.229	-0.081*	1.495	0.820	0.836		0.809	1.589
**Equation 5**	-28.670	0.055	0.211	0.229	-0.081*	1.503	0.796	0.844	-0.102*	0.808	1.594
**Men (n = 49)**											
**Equation 1**	-2.408	0.004	0.070	0.327	-0.110*					0.805	1.320
**Equation 2**	-1.761	0.003	0.067	0.319	-0.100*	-0.335*				0.802	1.329
**Equation 3**	-1.877	0.003	0.067	0.322	-0.101*	-0.346*	0.145			0.798	1.343
**Equation 4**	-4.248	0.014	0.073	0.332	-0.125*	-0.056*	-0.037*	1.850		0.807	1.312
**Equation 5**	-3.030	0.007	0.064	0.332	-0.121*	-0.156*	0.093	1.846	0.576	0.811	1.301
**Women (n = 106)**											
**Equation 1**	-6.828	0.008	0.085	0.196	-0.036*					0.726	1.198
**Equation 2**	-9.362	0.005	0.094	0.185	-0.029*	1.827				0.732	1.184
**Equation 3**	-8.948	0.006	0.091	0.182	-0.025*	1.776	-0.630*			0.732	1.183
**Equation 4**	-9.267	0.010	0.091	0.181	-0.025*	1.750	-0.648*	0.247		0.732	1.184
**Equation 5**	-9.302	0.011	0.091	0.181	-0.025*	1.785	-0.649*	0.255	-0.059*	0.729	1.190

*Denotes statistical significance (*P*<0.05)

Acronym: SEE, standard error of estimate

### Evaluation of the prediction equations with concordance correlation coefficient

Further evaluation of the performance among prediction equations with CCC ranged from 0.897 to 0.905 for BFM, from 0.838 to 0.856 for LBM, from 0.844 to 0.858 for TFM, and 0.876 to 0.888, respectively (**Table 6**). In general, the model performance was the best for FBM in men and LBM for women with the simplest equation (age, height, weight, waist and circumference).

**Table 6:** Concordance correlation coefficient for anthropometric prediction equations of body fat mass, lean body mass, trunk fat mass, and appendicular lean mass in the community-dwelling cancer survivors derived the Korea National Health and Nutrition Examination Survey (2008–2011).

CCC	Body fat mass	Lean body mass	Trunk fat mass	Appendicular lean mass
**Total(n = 155)**				
**Equation 1**	0.897	0.838	0.844	0.876
**Equation 2**	0.904	0.851	0.851	0.885
**Equation 3**	0.908	0.856	0.857	0.888
**Equation 4**	0.905	0.856	0.858	0.883
**Equation 5**	0.905	0.855	0.857	0.883
**Men(n = 49)**				
**Equation 1**	0.855	0.774	0.794	0.817
**Equation 2**	0.848	0.768	0.788	0.811
**Equation 3**	0.854	0.779	0.799	0.814
**Equation 4**	0.850	0.777	0.798	0.796
**Equation 5**	0.838	0.760	0.785	0.787
**Women(n = 106)**				
**Equation 1**	0.840	0.902	0.890	0.802
**Equation 2**	0.832	0.899	0.890	0.795
**Equation 3**	0.828	0.898	0.886	0.792
**Equation 4**	0.827	0.898	0.885	0.785
**Equation 5**	0.827	0.898	0.882	0.785

Acronym: CCC, concordance correlation coefficient

### Stratification by obesity status

Stratifying the study participants based on obesity status showed limited statistical power for predicting body composition, particularly for individuals with obesity. This limitation may be attributed to the relatively small sample size in this specific category, potentially influencing the precision and generalizability of our findings. S5–S12 Tables outlines the prediction equations for BFM, LBM, TFM, and ALM stratified by obesity status. The performance evaluation of these prediction equations in study participants, categorized by obesity status, is visually depicted through box plots (**S2 and S3 Figs**), Bland-Altman plots (**S4 and S5 Figs**), and CCC (**S13 and S14 Tables**).

### Comparison of performance to prediction equations developed in the general population

Further evaluation of the performance by comparing our equations with general population equations for BFM and LBM. Specifically, We compared equation 2 from Lee et al. (2017) and equation 1 from Lee et al. (2021), which include the same variables as the simplest equation (age, height, weight, waist and circumference) in our study. Our equation showed better predictive ability, with higher CCC and R^2^ values and lower SEE in both sexes compared to the general population equation **(S15 and S16 Tables)**.

## Discussion

This study developed equations for estimating body composition comprised of LBM, BFM, ALM, and TFM among the community-dwelling cancer survivors in the KNAHNES. Our equations were specifically designed to account for changes in distribution of body composition after cancer diagnosis potentially due to treatment-related complications and changes in lifestyle. Although several studies have developed anthropometric prediction equations for body composition, none have specifically addressed cancer survivors. Our study contributes meaningful enhancements to the existing pool of prediction equations for general population body composition, rendering it particularly pertinent for utilization in epidemiological research focused on cancer survivors.

Our results were generally comparable to the previous studies that used anthropometric data to develop predicted values of body composition from the U.S. NHANES and KNHANES.

Lee *et al*., developed body composition prediction equations for estimating lean body mass and body fat mass from a large sample dataset from the U.S. NHANES (1999–2006) including 14,065 participants (7,531 males and 6,534 females). In that study, the equations utilize variables such as age, race, height, weight, and waist circumference with high coefficient of determination (fat mass: *R*^2^ = 0.90, SEE = 2.6kg for male and *R*^2^ = 0.93, SEE = 2.4kg for female; lean body mass: *R*^2^ = 0.91, SEE = 2.6kg for male, *R*^2^ = 0.85, SEE = 2.4kg for female). In that study, waist circumference was a significant factor only for males. However, participants of the U.S. NHANES with history of cancer were not specifically addressed [22]. Similarly, another study utilized a sample dataset of 17,608 participants (7,599 males and 10,009 females) from the KNHANES (2008–2011) to develop a body measurement equation capable of predicting lean body mass and body fat mass using as age, height, weight, waist circumference, and factors influencing body composition, including smoking status, alcohol consumption, physical activity, and serum creatinine concentration. This prediction equation demonstrated the highest predictive ability for lean body mass(male: *R*^2^ = 0.85, SEE = 2.7kg, female: *R*^2^ = 0.78, SEE = 2.2kg) For body fat mass (BFM), the equation is as follows (male: *R*^2^ = 0.74, SEE = 2.7kg, female: *R*^2^ = 0.83, SEE = 2.2kg) [23]. Furthermore, all equations exhibited low bias and demonstrated high interclass correlation coefficient values. These outcomes underscore the potential applicability of such body measurement equations in various epidemiological studies. Also, Costa *et al*., developed and cross-validated prediction equations for fat-free mass and lean soft tissue mass with age, weight, height, waist circumference, and resistance in bioimpedance among young and middle-aged Brazilian women (R^2^ = 0.86 for fat-free mass and R^2^ = 0.92 for lean soft tissue) [29]. While bioelectric impedance could serve as a simple and non-invasive method for assessing physicological characterstics that would be of additional value for development of prediction equations for body composition, this data was not available in the KNHANES.

Moreover, recent systematic reviews have revealed variations in predictions based on age, weight status, or race/ethnicity within the population groups [30]. In this study, regardless of predictability or utilized variables, the equation is particularly well-suited for large cancer survivor cohorts due to notable disparities, especially in weight status, among cancer survivors. Unintentional weight loss after cancer diagnosis differs across cancer types, often being prominent in lung and gastrointestinal cancers. This phenomenon is influenced by a spectrum of factors including tumor response, metabolic changes due to inflammation, and other multifaceted factors [31, 32]. Specifically, in this study, there was a difference in predictability between men and women. This is because, even with the same BMI unit, women tend to have higher WC and BMI compared to men. Therefore, when conducting research on obesity, it is important to take these differences into consideration.

Notably, two investigators have used prediction equations for body composition to investigate the association of predicted fat mass and lean mass with all-cause mortality and incident CVD in epidemiological studies. Lee *et al*., applied prediction equation derived from the U.S. NHANES to examine association of predicted fat mass and predicted lean mass with mortality among the participants of the Health Professionals Follow-Up Study (HPFS) [25]. Particularly in male participants, low BMI in the lower group (BMI < 18.5 kg/m^2^) was associated with lower lean mass rather than lower fat mass suggests evidence for an increased mortality rate, indicating the potential relevance of lean mass in the context of mortality. However, the HPFS study excluded individuals with a history of cancer. This is because the presence of cancer history can lead to higher mortality rates, and also because the study aimed to account for weight changes attributed to cancer (i.e., low BMI associated with cancer). Similarly, another study used equation developed from the KNAHNES was applied to National Health Insurance Service-Health Screening Cohort (NHIS-HEALS) database to assess association between predict body fat mass, lean body mass, and appendicular muscle mass with future CVD risk [24]. The increase in predicted fat mass as well as decrease in predicted muscle mass were found to be associated with a significantly increased risk of incident CVD. However, even in the NHIS-HEALS study, cancer survivors were not directly addressed.

As cancer survivors have a higher risk of CVD compared to the general population [33] and may experience changes in body composition due to cancer-related therapies, it would be of clinical importance to develop specific prediction equations for body composition among cancer survivors. Findings from the US Surveillance, Epidemiology, and End Results data showed that cancer patients had a 2.17-fold increased risk of stroke as compared to those without cancer [34]. Also, utilizing the same equation as the general population for predicted fat mass and predicted lean mass among cancer survivors might lead to overestimation or underestimation due to weight changes after cancer diagnosis [31, 32]. There are also studies that use waist circumference (WC) as an indicator of adiposity instead of BMI. Research has shown that breast cancer survivors with higher WC had higher rates of cancer recurrence and mortality [35]. However, in studies that solely utilize WC as an indicator, the consideration of lean mass is not possible. In our study, we not only present predictive equations for estimated fat mass, a crucial indicator of adiposity, but also provide equations for estimated lean mass, allowing for a comprehensive assessment of predicted body composition for adult cancer survivors. Particularly, predicted lean mass could serve as an indicator for sarcopenia, the age-related progressive loss of muscle mass and strength, among cancer survivors [32]. Therefore, these equations could be applicable to large cancer survivor cohorts to investigate various health outcomes comprehensively. Additionally, this study notably incorporates factors related to sarcopenia in cancer survivors, as highlighted in the previous study with KNAHNES. Given that cancer survivors constitute a specific group known to have different body composition distributions compared to the general population, deriving predicted fat mass and lean mass values holds significance.

### Strengths and limitations

Our study has a few limitations that need to be considered. First, due to the limited availability of DXA measurements in the KNHANES in a certain period (2008–2011), only internal validation was feasible as collection of DXA data beyond 2011 was discontinued. Thus, further external validation for the prediction equations developed in this study with most up-to-date data is necessary. Moreover, since the sampling method (i.e., complex, stratified, and multistage probability sampling) of the KNHANES is designed for a representative selection of the entire population instead of cancer survivors, such method only encompasses the general population. Thus, the available sample size of cancer survivors with DXA data was relatively small and limited statistical power for further stratification by obesity status. To address the possible variation related to obesity, future studies should implement stratification by obesity status in development and cross-validation of the prediction equations for body composition.

Also, we were not able to incorporate specific variables such as primary cancer site, cancer stage at diagnosis, and time since diagnosis into the model development owing to the limited sample size (n = 155). Therefore, future studies with large sample size with information on such variables are warranted to account for potential variations in body composition by distinct characteristics of cancer survivors. Second, the KNHANES consist of a population primarily of a single ethnic group (i.e., Korean descendants) and thus the equation is predominantly applicable to Korean cancer survivors. Of note, a recent systematic review of existing body composition equations emphasized the importance of considering inter-ethnic differences alongside age and weight status [30, 36]. Consequently, when developing such equations for cancer survivors, utilizing a multi-ethnic cohort, if feasible, would likely enhance the representativeness and applicability. Third, the limits of agreement (LOA) found in this study varied by types of predicted body composition and by sex, which implies the potential need for training and standardization of prediction equations with larger samples. Lastly, it is noteworthy to acknowledge the potential bias in prediction excessive BFM and LBM, exemplified by values exceeding 7.0 kg in Bland-Altman plots. This bias may limit the applicability of the prediction equations for individuals with outlier values for BFM and LBM. This limitation underlines the need for cautious application of the prediction equations for body composition at individual levels. Despite these limitations, this study introduces a novel equation for estimating fat mass and lean mass specifically tailored to community-dwelling cancer survivors. The current study highlights the need for equations that can comprehensively assess fat mass and lean mass in cancer survivors, distinct from the general population. Importantly, the developed equations exhibited compatibility in terms of predictability.

## Conclusion

This study aimed to develop and validate prediction equations for LBM, BFM, ALM, and TFM using age, height, weight, waist circumference, serum creatinine levels, and lifestyle factors as independent variables. The values predicted using the developed equations did not significantly differ from those directly measured using DXA. This finding suggests the potential applicability of these equations to other epidemiological studies involving cancer survivors. Future studies should include a multi-ethnic cohort to account for population-specific differences in body composition.

## Supporting information

S1 Fig Bland-Altman plots for evaluating differences in actual and predicted values of trunk fat mass (TFM) and appendicular lean mass (ALM) in adult cancer survivors in the Korean National Health and Nutrition Examination Survey (2008–2011). (PPTX)

S2 Fig Boxplots comparing actual and predicted values of body fat mass and lean body mass in adult cancer Survivors with obesity (body mass index≥25.0 kg/m^2^) in the Korean National Health and Nutrition Examination Survey (2008–2011). (PPTX)

S3 Fig Boxplots comparing actual and predicted values of body fat mass and lean body mass in adult cancer survivors without obesity—(body mass index<25.0 kg/m^2^) in the Korean National Health and Nutrition Examination Survey (2008–2011). (PPTX)

S4 Fig Bland-Altman plots for evaluating differences in actual and predicted values of body fat mass (BFM) and lean body mass (LBM) in adult cancer survivors with obesity (body mass index≥25.0 kg/m^2^)in the Korean National Health and Nutrition Examination Survey (2008–2011). (PPTX)

S5 Fig Bland-Altman plots for evaluating differences in actual and predicted values of body fat mass (BFM) and lean body mass (LBM) in adult cancer survivors without obesity (body mass index<25.0 kg/m^2^)in the Korean National Health and Nutrition Examination Survey (2008–2011). (PPTX)

S1 Table Validation of anthropometric prediction equations for Fat body mass in the community-dwelling cancer survivors derived the Korea National Health and Nutrition Examination Survey (2008–2011). (DOCX)

S2 Table Validation of anthropometric prediction equations for lean body mass in the community-dwelling cancer survivors derived the Korea National Health and Nutrition Examination Survey (2008–2011). (DOCX)

S3 Table Validation of anthropometric prediction equations for trunk fat body mass in the community-dwelling cancer survivors derived the Korea National Health and Nutrition Examination Survey (2008–2011). (DOCX)

S4 Table Validation of anthropometric prediction equations for appendicular lean body mass in the community-dwelling cancer survivors derived the Korea National Health and Nutrition Examination Survey (2008–2011). (DOCX)

S5 Table Anthropometric prediction equations for body fat mass in the community-dwelling cancer survivors with obesity (body mass index≥25.0 kg/m^2^) derived the Korea National Health and Nutrition Examination Survey (2008–2011). (DOCX)

S6 Table Anthropometric prediction equations for lean body mass in the community-dwelling cancer survivors with obesity (body mass index≥25.0 kg/m^2^) derived the Korea National Health and Nutrition Examination Survey (2008–2011). (DOCX)

S7 Table Anthropometric prediction equations for trunk fat mass in the community-dwelling cancer survivors with obesity (body mass index≥25.0 kg/m^2^) derived the Korea National Health and Nutrition Examination Survey (2008–2011). (DOCX)

S8 Table Anthropometric prediction equations for appendicular lean mass in the community-dwelling cancer survivors with obesity (body mass index≥25.0 kg/m^2^) derived the Korea National Health and Nutrition Examination Survey (2008–2011). (DOCX)

S9 Table Anthropometric prediction equations for body fat mass in the community-dwelling cancer survivors without obesity (body mass index<25.0 kg/m^2^) derived the Korea National Health and Nutrition Examination Survey (2008–2011). (DOCX)

S10 Table Anthropometric prediction equations for lean body mass in the community-dwelling cancer survivors without obesity (body mass index<25.0 kg/m^2^) derived the Korea National Health and Nutrition Examination Survey (2008–2011). (DOCX)

S11 Table Anthropometric prediction equations for trunk fat mass in the community-dwelling cancer survivors without obesity (body mass index<25.0 kg/m^2^) derived the Korea National Health and Nutrition Examination Survey (2008–2011). (DOCX)

S12 Table Anthropometric prediction equations for appendicular lean mass in the community-dwelling cancer survivors without obesity (body mass index<25.0 kg/m^2^) derived the Korea National Health and Nutrition Examination Survey (2008–2011). (DOCX)

S13 Table Concordance correlation coefficient for anthropometric prediction equations of body fat mass, lean body mass, trunk fat mass, and appendicular lean mass in the community-dwelling cancer survivors with obesity (body mass index≥25.0 kg/m^2^) derived the Korea National Health and Nutrition Examination Survey (2008–2011). (DOCX)

S14 Table Concordance correlation coefficient for anthropometric prediction equations of body fat mass, lean body mass, trunk fat mass, and appendicular lean mass in the community-dwelling cancer survivors without obesity (body mass index<25.0 kg/m^2^) derived the Korea National Health and Nutrition Examination Survey (2008–2011). (DOCX)

S15 Table Comparison of performance to prediction equations developed in the general population for body fat mass in the cross-validation set. (DOCX)

S16 Table Comparison of performance to prediction equations developed in the general population for lean body mass in the cross-validation set. (DOCX)
