# Supplementary material for: Development and cross-validation of prediction equations for body composition in adult cancer survivors from the Korean National Health and Nutrition Examination Survey (KNHANES)
Source: PLoS One. 2024 Oct 4;19(10):e0309061. doi: 10.1371/journal.pone.0309061 (PMC11451997; doi:10.1371/journal.pone.0309061)
Supplement: S1 Fig — (PPTX) [file pone.0309061.s001.pptx]

## Slide 1
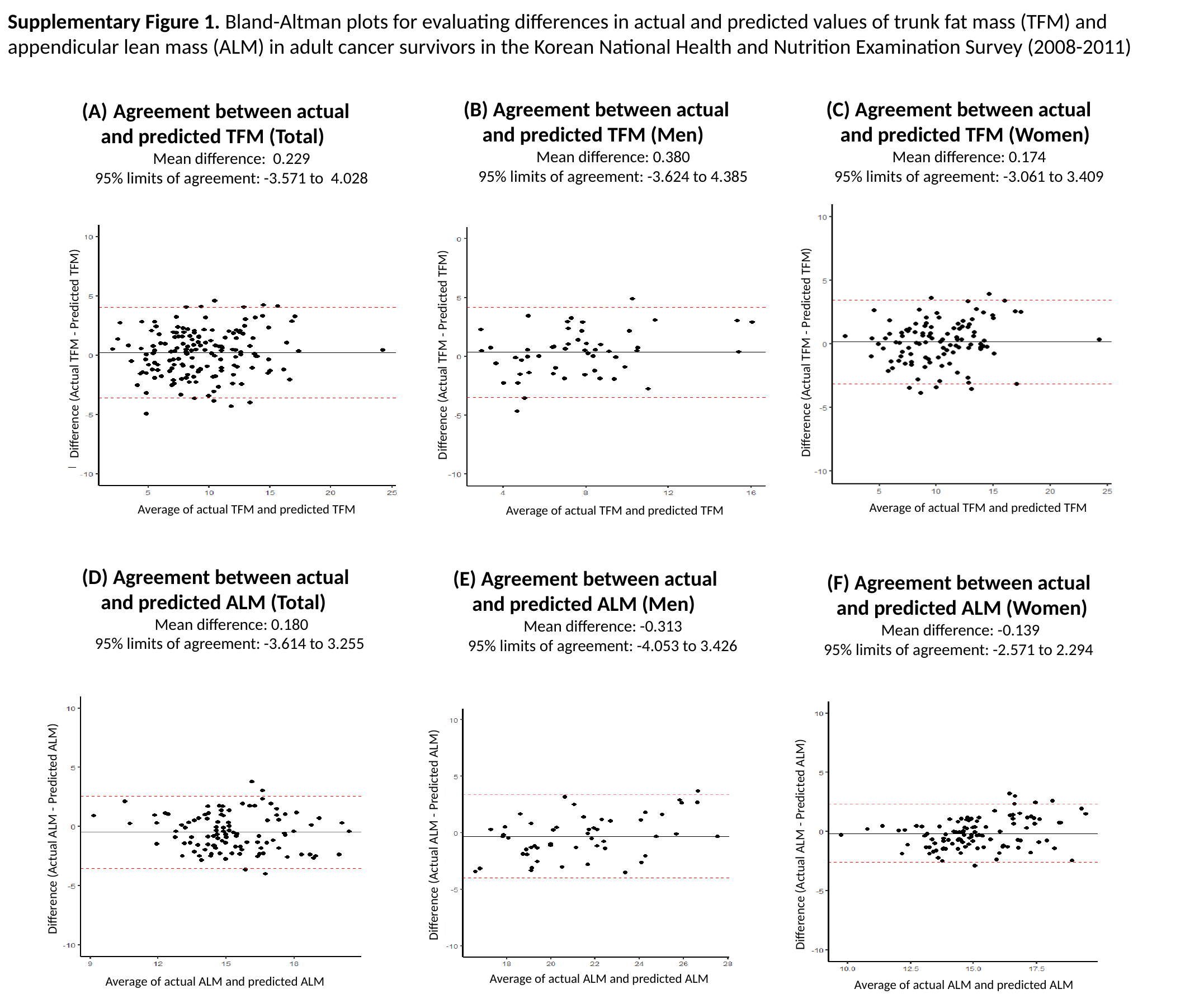

Supplementary Figure 1. Bland-Altman plots for evaluating differences in actual and predicted values of trunk fat mass (TFM) and appendicular lean mass (ALM) in adult cancer survivors in the Korean National Health and Nutrition Examination Survey (2008-2011)
(B) Agreement between actual
 and predicted TFM (Men)
Mean difference: 0.380
95% limits of agreement: -3.624 to 4.385
 (C) Agreement between actual
 and predicted TFM (Women)
Mean difference: 0.174
95% limits of agreement: -3.061 to 3.409
Agreement between actual
 and predicted TFM (Total)
Mean difference: 0.229
95% limits of agreement: -3.571 to 4.028
Difference (Actual TFM - Predicted TFM)
Difference (Actual TFM - Predicted TFM)
Difference (Actual TFM - Predicted TFM)
Difference (Actual TFM - Predicted TFM)
Difference (Actual TFM - Predicted TFM)
 Average of actual TFM and predicted TFM
 Average of actual TFM and predicted TFM
 Average of actual TFM and predicted TFM
(D) Agreement between actual
 and predicted ALM (Total)
Mean difference: 0.180
95% limits of agreement: -3.614 to 3.255
(E) Agreement between actual
 and predicted ALM (Men)
Mean difference: -0.313
95% limits of agreement: -4.053 to 3.426
 (F) Agreement between actual
 and predicted ALM (Women)
Mean difference: -0.139
95% limits of agreement: -2.571 to 2.294
 Difference (Actual ALM - Predicted ALM)
 Difference (Actual ALM - Predicted ALM)
 Difference (Actual ALM - Predicted ALM)
 Average of actual ALM and predicted ALM
 Average of actual ALM and predicted ALM
 Average of actual ALM and predicted ALM
