# Supplementary material for: Development and cross-validation of prediction equations for body composition in adult cancer survivors from the Korean National Health and Nutrition Examination Survey (KNHANES)
Source: PLoS One. 2024 Oct 4;19(10):e0309061. doi: 10.1371/journal.pone.0309061 (PMC11451997; doi:10.1371/journal.pone.0309061)
Supplement: S3 Fig — (PPTX) [file pone.0309061.s003.pptx]

## Slide 1
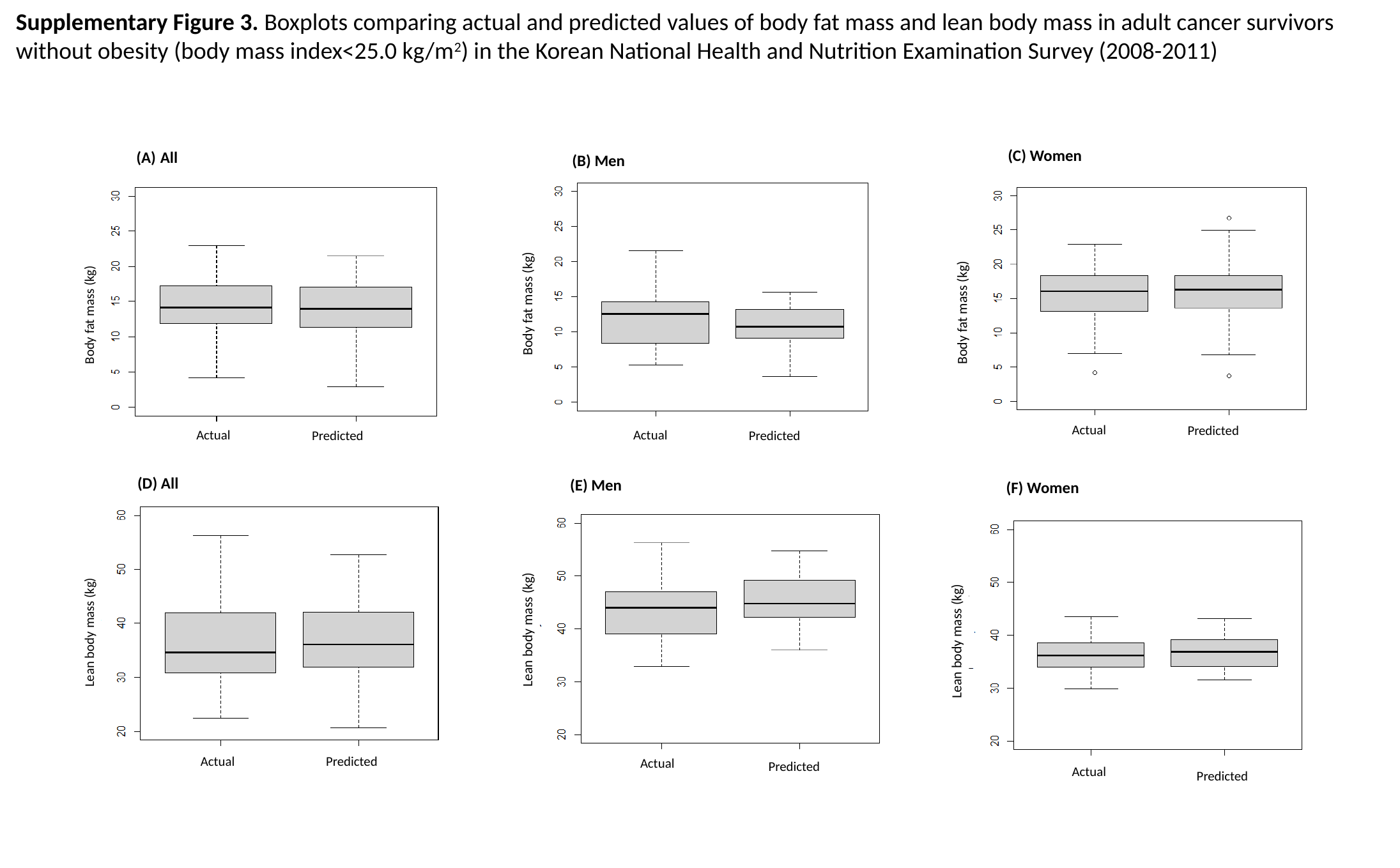

Supplementary Figure 3. Boxplots comparing actual and predicted values of body fat mass and lean body mass in adult cancer survivors without obesity (body mass index<25.0 kg/m2) in the Korean National Health and Nutrition Examination Survey (2008-2011)
(C) Women
All
(B) Men
Body fat mass (kg)
Body Fat mass
Body Fat mass
Body fat mass (kg)
Body fat mass (kg)
Actual
Predicted
Actual
Actual
Predicted
Predicted
(D) All
(E) Men
(F) Women
Lean body mass
Lean body mass
Lean body mass (kg)
Lean body mass (kg)
Lean body mass (kg)
Actual
Predicted
Actual
Predicted
Actual
Predicted
