# Supplementary material for: Development and cross-validation of prediction equations for body composition in adult cancer survivors from the Korean National Health and Nutrition Examination Survey (KNHANES)
Source: PLoS One. 2024 Oct 4;19(10):e0309061. doi: 10.1371/journal.pone.0309061 (PMC11451997; doi:10.1371/journal.pone.0309061)
Supplement: S4 Fig — (PPTX) [file pone.0309061.s004.pptx]

## Slide 1
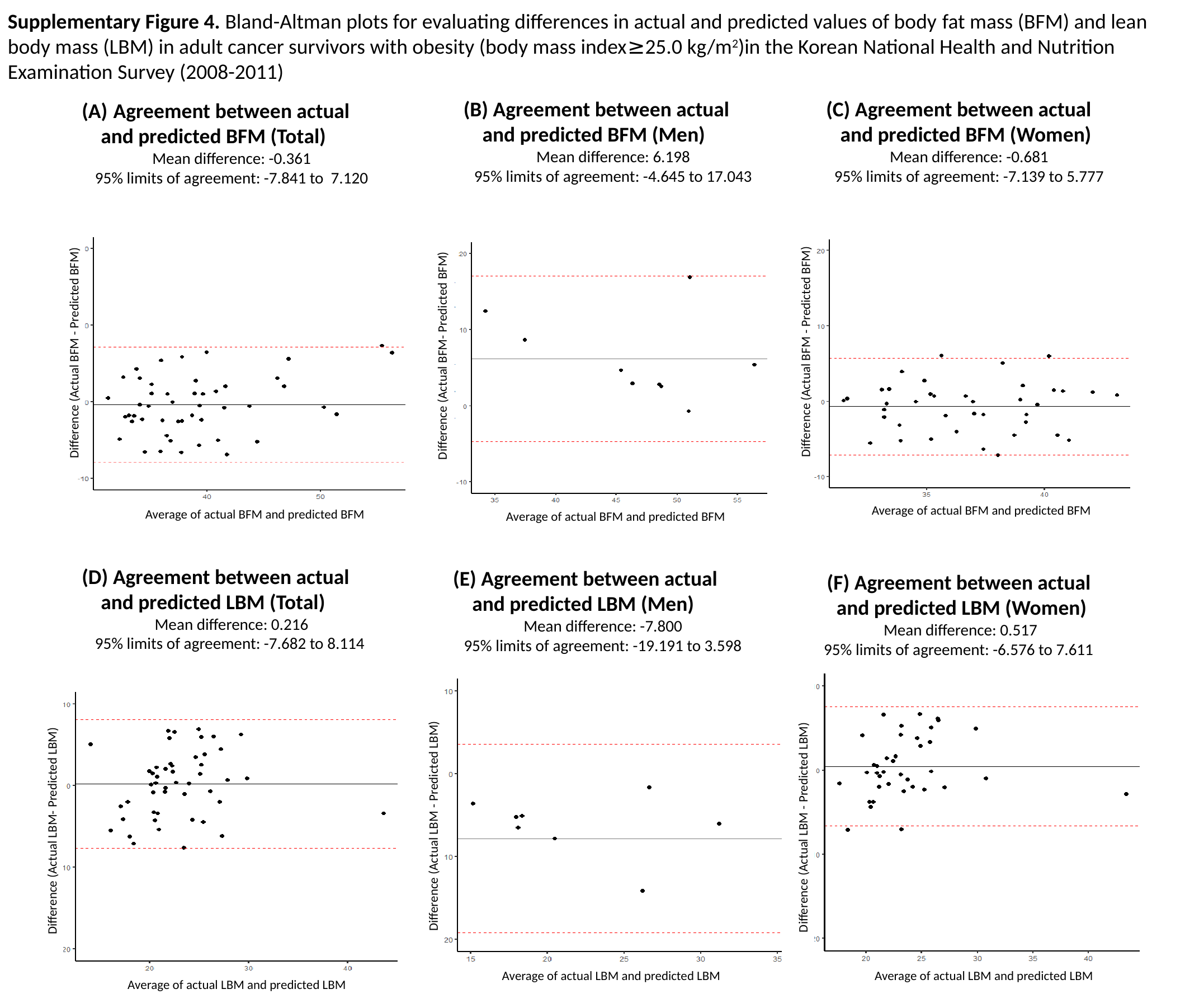

Supplementary Figure 4. Bland-Altman plots for evaluating differences in actual and predicted values of body fat mass (BFM) and lean body mass (LBM) in adult cancer survivors with obesity (body mass index≥25.0 kg/m2)in the Korean National Health and Nutrition Examination Survey (2008-2011)
(B) Agreement between actual
 and predicted BFM (Men)
Mean difference: 6.198
95% limits of agreement: -4.645 to 17.043
 (C) Agreement between actual
 and predicted BFM (Women)
Mean difference: -0.681
95% limits of agreement: -7.139 to 5.777
Agreement between actual
 and predicted BFM (Total)
Mean difference: -0.361
95% limits of agreement: -7.841 to 7.120
Difference (Actual TFM - Predicted TFM)
Difference (Actual BFM - Predicted BFM)
Difference (Actual BFM - Predicted BFM)
Difference (Actual TFM - Predicted TFM)
Difference (Actual BFM- Predicted BFM)
 Average of actual BFM and predicted BFM
 Average of actual BFM and predicted BFM
 Average of actual BFM and predicted BFM
(D) Agreement between actual
 and predicted LBM (Total)
Mean difference: 0.216
95% limits of agreement: -7.682 to 8.114
(E) Agreement between actual
 and predicted LBM (Men)
Mean difference: -7.800
95% limits of agreement: -19.191 to 3.598
 (F) Agreement between actual
 and predicted LBM (Women)
Mean difference: 0.517
95% limits of agreement: -6.576 to 7.611
 Difference (Actual LBM - Predicted LBM)
 Difference (Actual LBM - Predicted LBM)
 Difference (Actual LBM- Predicted LBM)
 Average of actual LBM and predicted LBM
 Average of actual LBM and predicted LBM
 Average of actual LBM and predicted LBM
