# Supplementary material for: Development and cross-validation of prediction equations for body composition in adult cancer survivors from the Korean National Health and Nutrition Examination Survey (KNHANES)
Source: PLoS One. 2024 Oct 4;19(10):e0309061. doi: 10.1371/journal.pone.0309061 (PMC11451997; doi:10.1371/journal.pone.0309061)
Supplement: S5 Fig — (PPTX) [file pone.0309061.s005.pptx]

## Slide 1
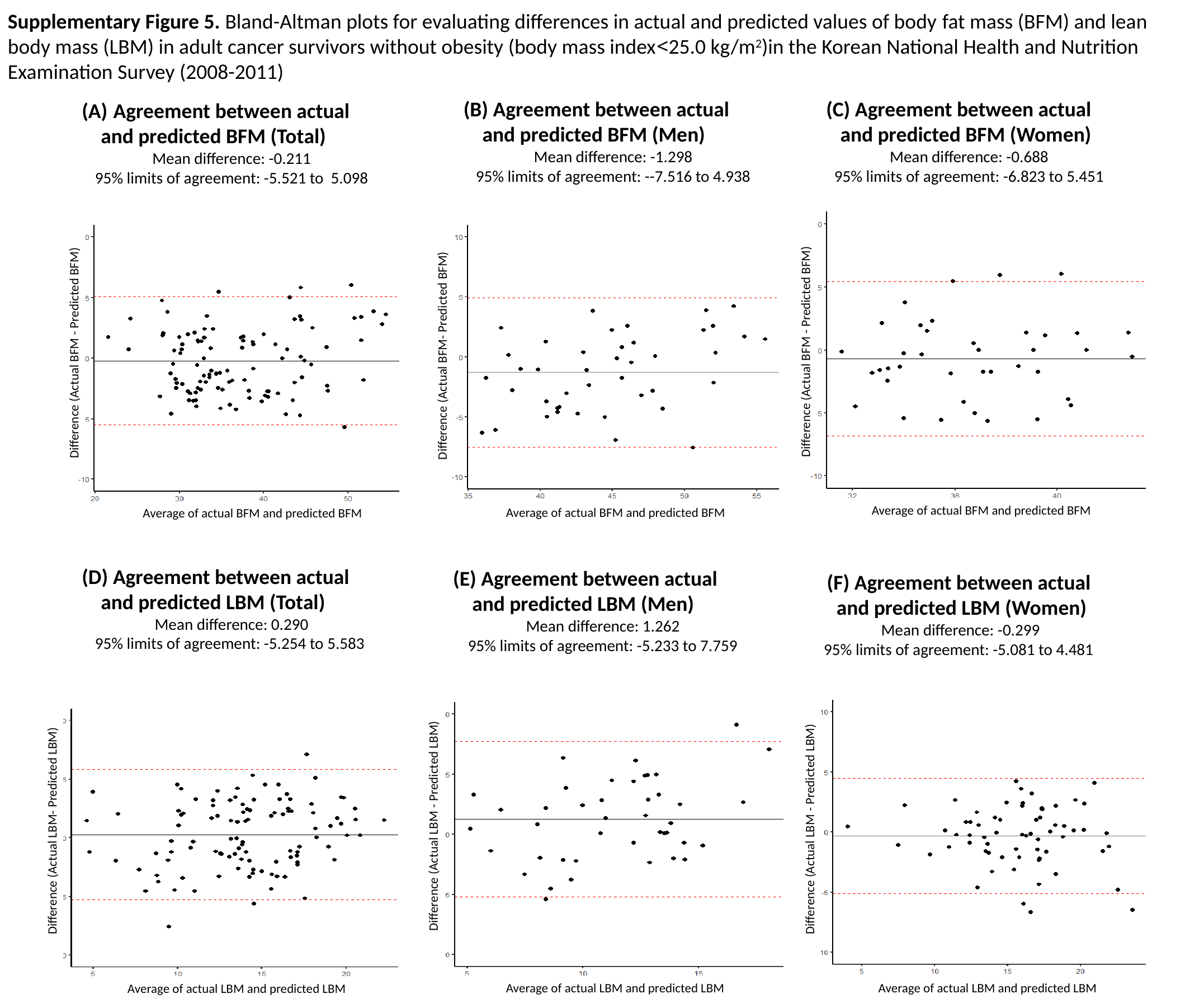

Supplementary Figure 5. Bland-Altman plots for evaluating differences in actual and predicted values of body fat mass (BFM) and lean body mass (LBM) in adult cancer survivors without obesity (body mass index<25.0 kg/m2)in the Korean National Health and Nutrition Examination Survey (2008-2011)
(B) Agreement between actual
 and predicted BFM (Men)
Mean difference: -1.298
95% limits of agreement: --7.516 to 4.938
 (C) Agreement between actual
 and predicted BFM (Women)
Mean difference: -0.688
95% limits of agreement: -6.823 to 5.451
Agreement between actual
 and predicted BFM (Total)
Mean difference: -0.211
95% limits of agreement: -5.521 to 5.098
Difference (Actual TFM - Predicted TFM)
Difference (Actual BFM - Predicted BFM)
Difference (Actual BFM - Predicted BFM)
Difference (Actual TFM - Predicted TFM)
Difference (Actual BFM- Predicted BFM)
 Average of actual BFM and predicted BFM
 Average of actual BFM and predicted BFM
 Average of actual BFM and predicted BFM
(D) Agreement between actual
 and predicted LBM (Total)
Mean difference: 0.290
95% limits of agreement: -5.254 to 5.583
(E) Agreement between actual
 and predicted LBM (Men)
Mean difference: 1.262
95% limits of agreement: -5.233 to 7.759
 (F) Agreement between actual
 and predicted LBM (Women)
Mean difference: -0.299
95% limits of agreement: -5.081 to 4.481
 Difference (Actual LBM - Predicted LBM)
 Difference (Actual LBM - Predicted LBM)
 Difference (Actual LBM- Predicted LBM)
 Average of actual LBM and predicted LBM
 Average of actual LBM and predicted LBM
 Average of actual LBM and predicted LBM
