# Supplementary material for: Development and cross-validation of prediction equations for body composition in adult cancer survivors from the Korean National Health and Nutrition Examination Survey (KNHANES)
Source: PLoS One. 2024 Oct 4;19(10):e0309061. doi: 10.1371/journal.pone.0309061 (PMC11451997; doi:10.1371/journal.pone.0309061)
Supplement: S1 Table — (DOCX) [file pone.0309061.s006.docx]

**Supplementary Table 1.** Validation of anthropometric prediction equations for Fat body mass in the community-dwelling cancer survivors derived the Korea National Health and Nutrition Examination Survey (2008-2011)

| Total fat mass | Difference | SD | $\boldsymbol{p}_{\boldsymbol{paired t test}}$ | $\boldsymbol{R}^{\boldsymbol{2}}$ | SEE |
| --- | --- | --- | --- | --- | --- |
| Total(n=155) |  |  |  |  |  |
| Equation 1 | -0.23 | 0.25 | 0.35 | 0.819 | 3.124 |
| Equation 2 | -0.20 | 0.24 | 0.41 | 0.827 | 3.060 |
| Equation 3 | -0.16 | 0.24 | 0.50 | 0.829 | 3.036 |
| Equation 4 | -0.17 | 0.25 | 0.49 | 0.828 | 3.045 |
| Equation 5 | -0.17 | 0.25 | 0.49 | 0.829 | 3.040 |
| Equation 6 | -0.23 | 0.26 | 0.38 | 0.818 | 3.134 |
| Men(n=51) |  |  |  |  |  |
| Equation 1 | -0.63 | 0.42 | 0.15 | 0.797 | 2.914 |
| Equation 2 | -0.64 | 0.43 | 0.15 | 0.794 | 2.933 |
| Equation 3 | -0.60 | 0.43 | 0.17 | 0.790 | 2.957 |
| Equation 4 | -0.61 | 0.43 | 0.16 | 0.793 | 2.939 |
| Equation 5 | -0.65 | 0.45 | 0.15 | 0.803 | 2.867 |
| Equation 6 | -0.68 | 0.45 | 0.14 | 0.802 | 2.877 |
| Women(n=104) |  |  |  |  |  |
| Equation 1 | 0.03 | 0.23 | 0.88 | 0.722 | 2.287 |
| Equation 2 | -0.01 | 0.23 | 0.97 | 0.719 | 2.298 |
| Equation 3 | 0.00 | 0.24 | 1.00 | 0.717 | 2.308 |
| Equation 4 | 0.00 | 0.24 | 1.00 | 0.714 | 2.319 |
| Equation 5 | -0.23 | 0.25 | 0.99 | 0.713 | 2.322 |
| Equation 6 | 0.04 | 0.23 | 0.87 | 0.719 | 2.299 |

Acronym: SEE, standard error of estimate
