# Supplementary material for: Development and cross-validation of prediction equations for body composition in adult cancer survivors from the Korean National Health and Nutrition Examination Survey (KNHANES)
Source: PLoS One. 2024 Oct 4;19(10):e0309061. doi: 10.1371/journal.pone.0309061 (PMC11451997; doi:10.1371/journal.pone.0309061)
Supplement: S2 Table — (DOCX) [file pone.0309061.s007.docx]

**Supplementary Table 2.** Validation of anthropometric prediction equations for lean body mass in the community-dwelling cancer survivors derived the Korea National Health and Nutrition Examination Survey (2008-2011)

| Total lean mass | Difference | SD | $\boldsymbol{p}_{\boldsymbol{paired t test}}$ | $\boldsymbol{R}^{\boldsymbol{2}}$ | SEE |
| --- | --- | --- | --- | --- | --- |
| Total(n=155) |  |  |  |  |  |
| Equation 1 | 0.27 | 0.26 | 0.31 | 0.710 | 3.304 |
| Equation 2 | 0.24 | 0.26 | 0.36 | 0.724 | 3.222 |
| Equation 3 | 0.19 | 0.25 | 0.46 | 0.732 | 3.175 |
| Equation 4 | 0.19 | 0.26 | 0.45 | 0.731 | 3.183 |
| Equation 5 | 0.19 | 0.26 | 0.45 | 0.731 | 3.183 |
| Equation 6 | 0.27 | 0.27 | 0.33 | 0.708 | 3.314 |
| Men(n=51) |  |  |  |  |  |
| Equation 1 | 0.66 | 0.44 | 0.14 | 0.634 | 3.070 |
| Equation 2 | 0.67 | 0.45 | 0.14 | 0.631 | 3.081 |
| Equation 3 | 0.60 | 0.44 | 0.18 | 0.630 | 3.084 |
| Equation 4 | 0.61 | 0.44 | 0.17 | 0.634 | 3.069 |
| Equation 5 | 0.66 | 0.47 | 0.16 | 0.647 | 3.015 |
| Equation 6 | 0.73 | 0.47 | 0.13 | 0.636 | 3.061 |
| Women(n=104) |  |  |  |  |  |
| Equation 1 | -0.01 | 0.24 | 0.96 | 0.824 | 2.449 |
| Equation 2 | 0.03 | 0.24 | 0.91 | 0.822 | 2.461 |
| Equation 3 | 0.02 | 0.25 | 0.94 | 0.821 | 2.469 |
| Equation 4 | 0.02 | 0.25 | 0.94 | 0.819 | 2.482 |
| Equation 5 | 0.02 | 0.25 | 0.95 | 0.818 | 2.489 |
| Equation 6 | -0.02 | 0.24 | 0.95 | 0.821 | 2.467 |

Acronym: SEE, standard error of estimate
