# Supplementary material for: Development and cross-validation of prediction equations for body composition in adult cancer survivors from the Korean National Health and Nutrition Examination Survey (KNHANES)
Source: PLoS One. 2024 Oct 4;19(10):e0309061. doi: 10.1371/journal.pone.0309061 (PMC11451997; doi:10.1371/journal.pone.0309061)
Supplement: S3 Table — (DOCX) [file pone.0309061.s008.docx]

**Supplementary Table 3**. Validation of anthropometric prediction equations for trunk fat body mass in the community-dwelling cancer survivors derived the Korea National Health and Nutrition Examination Survey (2008-2011)

| Trunk fat mass | Difference | SD | $\boldsymbol{p}_{\boldsymbol{paired t test}}$ | $\boldsymbol{R}^{\boldsymbol{2}}$ | SEE |
| --- | --- | --- | --- | --- | --- |
| Total(n=155) |  |  |  |  |  |
| Equation 1 | 0.27 | 0.16 | 0.10 | 0.718 | 2.010 |
| Equation 2 | 0.25 | 0.16 | 0.12 | 0.724 | 1.989 |
| Equation 3 | 0.23 | 0.16 | 0.15 | 0.733 | 1.956 |
| Equation 4 | 0.23 | 0.16 | 0.14 | 0.732 | 1.959 |
| Equation 5 | 0.23 | 0.16 | 0.14 | 0.732 | 1.962 |
| Equation 6 | 0.27 | 0.16 | 0.10 | 0.717 | 2.014 |
| Men(n=51) |  |  |  |  |  |
| Equation 1 | 0.39 | 0.28 | 0.17 | 0.661 | 1.980 |
| Equation 2 | 0.39 | 0.28 | 0.17 | 0.655 | 1.998 |
| Equation 3 | 0.35 | 0.27 | 0.21 | 0.655 | 1.996 |
| Equation 4 | 0.35 | 0.27 | 0.21 | 0.664 | 1.972 |
| Equation 5 | 0.38 | 0.29 | 0.19 | 0.666 | 1.966 |
| Equation 6 | 0.42 | 0.29 | 0.15 | 0.664 | 1.971 |
| Women(n=104) |  |  |  |  |  |
| Equation 1 | 0.17 | 0.16 | 0.29 | 0.793 | 1.673 |
| Equation 2 | 0.18 | 0.16 | 0.26 | 0.791 | 1.681 |
| Equation 3 | 0.17 | 0.17 | 0.30 | 0.791 | 1.684 |
| Equation 4 | 0.17 | 0.17 | 0.30 | 0.789 | 1.691 |
| Equation 5 | 0.16 | 0.17 | 0.33 | 0.788 | 1.696 |
| Equation 6 | 0.17 | 0.17 | 0.32 | 0.791 | 1.682 |

Acronym: SEE, standard error of estimate
