# Supplementary material for: Development and cross-validation of prediction equations for body composition in adult cancer survivors from the Korean National Health and Nutrition Examination Survey (KNHANES)
Source: PLoS One. 2024 Oct 4;19(10):e0309061. doi: 10.1371/journal.pone.0309061 (PMC11451997; doi:10.1371/journal.pone.0309061)
Supplement: S4 Table — (DOCX) [file pone.0309061.s009.docx]

**Supplementary Table 4.** Validation of anthropometric prediction equations for appendicular lean body mass in the community-dwelling cancer survivors derived the Korea National Health and Nutrition Examination Survey (2008-2011)

| Appendicular lean mass | Difference | SD | $\boldsymbol{p}_{\boldsymbol{paired t test}}$ | $\boldsymbol{R}^{\boldsymbol{2}}$ | SEE |
| --- | --- | --- | --- | --- | --- |
| Total(n=155) |  |  |  |  |  |
| Equation 1 | -0.21 | 0.14 | 0.14 | 0.787 | 1.784 |
| Equation 2 | -0.19 | 0.14 | 0.16 | 0.797 | 1.742 |
| Equation 3 | -0.18 | 0.14 | 0.20 | 0.799 | 1.732 |
| Equation 4 | -0.18 | 0.14 | 0.20 | 0.798 | 1.738 |
| Equation 5 | -0.18 | 0.14 | 0.20 | 0.797 | 1.740 |
| Equation 6 | -0.21 | 0.15 | 0.16 | 0.784 | 1.795 |
| Men(n=51) |  |  |  |  |  |
| Equation 1 | -0.28 | 0.25 | 0.27 | 0.708 | 1.796 |
| Equation 2 | -0.28 | 0.25 | 0.27 | 0.705 | 1.805 |
| Equation 3 | -0.28 | 0.25 | 0.28 | 0.700 | 1.821 |
| Equation 4 | -0.30 | 0.26 | 0.26 | 0.703 | 1.811 |
| Equation 5 | -0.31 | 0.27 | 0.25 | 0.714 | 1.779 |
| Equation 6 | -0.32 | 0.27 | 0.24 | 0.711 | 1.786 |
| Women(n=104) |  |  |  |  |  |
| Equation 1 | -0.13 | 0.12 | 0.28 | 0.662 | 1.223 |
| Equation 2 | -0.15 | 0.12 | 0.23 | 0.659 | 1.229 |
| Equation 3 | -0.14 | 0.12 | 0.26 | 0.656 | 1.234 |
| Equation 4 | -0.14 | 0.12 | 0.27 | 0.655 | 1.236 |
| Equation 5 | -0.14 | 0.12 | 0.26 | 0.652 | 1.241 |
| Equation 6 | -0.13 | 0.12 | 0.29 | 0.659 | 1.229 |

Acronym: SEE, standard error of estimate
