# Supplementary material for: Development and cross-validation of prediction equations for body composition in adult cancer survivors from the Korean National Health and Nutrition Examination Survey (KNHANES)
Source: PLoS One. 2024 Oct 4;19(10):e0309061. doi: 10.1371/journal.pone.0309061 (PMC11451997; doi:10.1371/journal.pone.0309061)
Supplement: S5 Table — (DOCX) [file pone.0309061.s010.docx]

**Supplementary Table 5**. Anthropometric prediction equations for body fat mass in the community-dwelling cancer survivors with obesity (body mass index≥25.0 kg/m^2^) derived the Korea National Health and Nutrition Examination Survey (2008-2011)

| Body fat mass |  |  |  |  |  |  |  |  |  |  |  |
| --- | --- | --- | --- | --- | --- | --- | --- | --- | --- | --- | --- |
|  | **Intercept** | **Age (years)** | **Height (cm)** | **Weight (kg)** | **Waist circumference (cm)** | **Creatinine**  **(mg/dL)** | **Smoking** | **Alcohol consumption** | **Physically inactive** | $\boldsymbol{R}^{\boldsymbol{2}}$ | **SEE** |
| Total(n=48) |  |  |  |  |  |  |  |  |  |  |  |
| Equation 1 | -74.648* | 0.084 | 0.579* | 0.276* | -0.004 |  |  |  |  | 0.838 | 2.654 |
| Equation 2 | -72.135* | 0.036 | 0.555* | 0.222* | 0.031 | 5.950* |  |  |  | 0.851 | 2.538 |
| Equation 3 | -68.366* | 0.036 | 0.540* | 0.246* | 0.004 | 5.051 | 1.529 |  |  | 0.853 | 2.521 |
| Equation 4 | -71.847* | 0.024 | 0.573* | 0.231* | 0.013 | 4.769 | 1.749 | -0.747 |  | 0.851 | 2.541 |
| Equation 5 | -73.070* | 0.015 | 0.571* | 0.231* | 0.026 | 5.125 | 1.977 | -1.154 | 1.020 | 0.851 | 2.544 |
| Equation 6 | -77.776* | 0.074 | 0.603* | 0.263* | 0.009 |  |  | -0.599 | 0.271 | 0.831 | 2.711 |
| Men(n=10) |  |  |  |  |  |  |  |  |  |  |  |
| Equation 1 | -23.870 | -0.076 | 0.580 | 0.163 | -0.335 |  |  |  |  | 0.666 | 2.721 |
| Equation 2 | -18.428 | -0.089 | 0.531 | 0.230 | -0.330 | -1.821 |  |  |  | 0.585 | 3.034 |
| Equation 3 | -47.593 | -0.235 | 0.763 | 0.284 | -0.245 | -14.682 | 3.956 |  |  | 0.607 | 2.950 |
| Equation 4 | -47.593 | -0.235 | 0.763 | 0.284 | -0.245 | -14.682 | 3.956 | 0.000 |  | 0.607 | 2.950 |
| Equation 5 | -87.644 | -0.292 | 1.142 | -0.107 | -0.280 | -7.212 | 6.298 | 0.000 | 4.987 | 0.809 | 2.056 |
| Equation 6 | -15.562 | -0.105 | 0.521 | 0.212 | -0.353 |  |  | 0.000 | 1.860 | 0.637 | 2.837 |
| Women(n=38) |  |  |  |  |  |  |  |  |  |  |  |
| Equation 1 | -42.500* | 0.039 | 0.358* | 0.338* | -0.004 |  |  |  |  | 0.811 | 2.112 |
| Equation 2 | -46.870* | 0.025 | 0.382* | 0.307* | 0.015 | 2.709 |  |  |  | 0.811 | 2.113 |
| Equation 3 | -49.383* | 0.027 | 0.393* | 0.295* | 0.032 | 2.649 | -0.894 |  |  | 0.806 | 2.138 |
| Equation 4 | -53.207* | 0.013 | 0.430* | 0.279* | 0.041 | 2.379 | -0.586 | -0.841 |  | 0.804 | 2.152 |
| Equation 5 | -55.967* | 0.005 | 0.427* | 0.275* | 0.075 | 2.403 | -1.049 | -1.382 | 1.507 | 0.811 | 2.113 |
| Equation 6 | -50.429* | 0.012 | 0.406* | 0.310* | 0.041 |  |  | -1.586 | 1.394 | 0.816 | 2.082 |

^*^Denotes statistical significance (*P*<0.05)

Acronym: SEE, standard error of estimate
