# Supplementary material for: Development and cross-validation of prediction equations for body composition in adult cancer survivors from the Korean National Health and Nutrition Examination Survey (KNHANES)
Source: PLoS One. 2024 Oct 4;19(10):e0309061. doi: 10.1371/journal.pone.0309061 (PMC11451997; doi:10.1371/journal.pone.0309061)
Supplement: S6 Table — (DOCX) [file pone.0309061.s011.docx]

**Supplementary Table 6**. Anthropometric prediction equations for lean body mass in the community-dwelling cancer survivors with obesity (body mass index≥25.0 kg/m^2^) derived the Korea National Health and Nutrition Examination Survey (2008-2011)

| Lean body mass |  |  |  |  |  |  |  |  |  |  |  |
| --- | --- | --- | --- | --- | --- | --- | --- | --- | --- | --- | --- |
|  | **Intercept** | **Age (years)** | **Height (cm)** | **Weight (kg)** | **Waist circumference (cm)** | **Creatinine**  **(mg/dL)** | **Smoking** | **Alcohol consumption** | **Physically inactive** | $\boldsymbol{R}^{\boldsymbol{2}}$ | **SEE** |
| Total(n=48) |  |  |  |  |  |  |  |  |  |  |  |
| Equation 1 | 80.307* | -0.080 | -0.637* | 0.714* | -0.013 |  |  |  |  | 0.740 | 2.947 |
| Equation 2 | 77.357* | -0.023 | -0.609* | 0.777* | -0.055 | -6.982* |  |  |  | 0.765 | 2.799 |
| Equation 3 | 72.153* | -0.023 | -0.587* | 0.743* | -0.017 | -5.741 | -2.112 |  |  | 0.774 | 2.749 |
| Equation 4 | 76.501* | -0.008 | -0.629* | 0.763* | -0.028 | -5.389 | -2.386 | 0.933 |  | 0.771 | 2.767 |
| Equation 5 | 77.807* | 0.001 | -0.626* | 0.763* | -0.043 | -5.770 | -2.630 | 1.368 | -1.090 | 0.770 | 2.771 |
| Equation 6 | 83.528* | -0.06 | -0.663* | 0.728* | -0.026 |  |  | 0.613 | -0.169 | 0.728 | 3.011 |
| Men(n=10) |  |  |  |  |  |  |  |  |  |  |  |
| Equation 1 | 13.636 | -0.020 | -0.512 | 0.764 | 0.384 |  |  |  |  | 0.418 | 3.048 |
| Equation 2 | 20.395 | -0.036 | -0.571 | 0.848 | 0.389 | -2.262 |  |  |  | 0.277 | 3.397 |
| Equation 3 | 57.708 | 0.151 | -0.868 | 0.779 | 0.280 | 14.192 | -5.062 |  |  | 0.402 | 3.090 |
| Equation 4 | 57.708 | 0.151 | -0.868 | 0.779 | 0.280 | 14.192 | -5.062 | 0.000 |  | 0.402 | 3.090 |
| Equation 5 | 103.100 | 0.216 | -1.297 | 1.222 | 0.320 | 5.725 | -7.716 | 0.000 | -5.652 | 0.813 | 1.726 |
| Equation 6 | 7.258 | 0.002 | -0.466 | 0.726 | 0.397 |  |  | 0.000 | -1.428 | 0.317 | 3.302 |
| Women(n=38) |  |  |  |  |  |  |  |  |  |  |  |
| Equation 1 | 42.744* | -0.019 | -0.385* | 0.651* | -0.016 |  |  |  |  | 0.807 | 2.328 |
| Equation 2 | 48.157* | -0.002 | -0.413* | 0.690* | -0.040 | -3.355 |  |  |  | 0.808 | 2.320 |
| Equation 3 | 51.363* | -0.004 | -0.428* | 0.705* | -0.061 | -3.279 | 1.141 |  |  | 0.804 | 2.344 |
| Equation 4 | 56.145* | 0.014 | -0.474* | 0.726* | -0.073 | -2.942 | 0.755 | 1.052 |  | 0.803 | 2.352 |
| Equation 5 | 59.230* | 0.022 | -0.471* | 0.730* | -0.111 | -2.968 | 1.273 | 1.656 | -1.683 | 0.811 | 2.306 |
| Equation 6 | 52.440* | 0.013 | -0.445* | 0.687* | -0.069 |  |  | 1.906 | -1.546 | 0.814 | 2.283 |

^*^Denotes statistical significance (*P*<0.05)

Acronym: SEE, standard error of estimate
