# Supplementary material for: Development and cross-validation of prediction equations for body composition in adult cancer survivors from the Korean National Health and Nutrition Examination Survey (KNHANES)
Source: PLoS One. 2024 Oct 4;19(10):e0309061. doi: 10.1371/journal.pone.0309061 (PMC11451997; doi:10.1371/journal.pone.0309061)
Supplement: S7 Table — (DOCX) [file pone.0309061.s012.docx]

**Supplementary Table 7**. Anthropometric prediction equations for trunk fat mass in the community-dwelling cancer survivors with obesity (body mass index≥25.0 kg/m^2^) derived the Korea National Health and Nutrition Examination Survey (2008-2011)

| Trunk fat mass |  |  |  |  |  |  |  |  |  |  |  |
| --- | --- | --- | --- | --- | --- | --- | --- | --- | --- | --- | --- |
|  | **Intercept** | **Age (years)** | **Height (cm)** | **Weight (kg)** | **Waist circumference (cm)** | **Creatinine**  **(mg/dL)** | **Smoking** | **Alcohol consumption** | **Physically inactive** | $\boldsymbol{R}^{\boldsymbol{2}}$ | **SEE** |
| Total(n=48) |  |  |  |  |  |  |  |  |  |  |  |
| Equation 1 | 29.826* | -0.017 | -0.329* | 0.367* | 0.122* |  |  |  |  | 0.739 | 1.801 |
| Equation 2 | 28.991* | -0.001 | -0.321* | 0.385* | 0.111 | -1.976 |  |  |  | 0.739 | 1.799 |
| Equation 3 | 25.384* | -0.001 | -0.306* | 0.362* | 0.137* | -1.115 | -1.464 |  |  | 0.751 | 1.759 |
| Equation 4 | 27.646* | 0.007 | -0.328* | 0.372* | 0.131* | -0.932 | -1.606 | 0.485 |  | 0.747 | 1.774 |
| Equation 5 | 29.055* | 0.016 | -0.325* | 0.373* | 0.115 | -1.343 | -1.869* | 0.955 | -1.176 | 0.756 | 1.739 |
| Equation 6 | 31.703* | -0.012 | -0.335* | 0.369* | 0.110 |  |  | 0.376 | -0.722 | 0.732 | 1.823 |
| Men(n=10) |  |  |  |  |  |  |  |  |  |  |  |
| Equation 1 | -0.289 | -0.018 | -0.233 | 0.371 | 0.265 |  |  |  |  | 0.143 | 2.440 |
| Equation 2 | -4.718 | -0.008 | -0.194 | 0.316 | 0.261 | 1.483 |  |  |  | -0.067 | 2.722 |
| Equation 3 | 29.529 | 0.164 | -0.466 | 0.252 | 0.160 | 16.584 | -4.646 |  |  | 0.286 | 2.227 |
| Equation 4 | 29.529 | 0.164 | -0.466 | 0.252 | 0.160 | 16.584 | -4.646 | 0.000 |  | 0.286 | 2.227 |
| Equation 5 | 61.208 | 0.209 | -0.766 | 0.562 | 0.188 | 10.675 | -6.498* | 0.000 | -3.945 | 0.724 | 1.384 |
| Equation 6 | -5.321 | -0.001 | -0.198 | 0.340 | 0.275 |  |  | 0.000 | -1.126 | -0.008 | 2.646 |
| Women(n=38) |  |  |  |  |  |  |  |  |  |  |  |
| Equation 1 | 0.490 | 0.031 | -0.142 | 0.324* | 0.132* |  |  |  |  | 0.837 | 1.382 |
| Equation 2 | -1.553 | 0.024 | -0.131 | 0.310* | 0.140* | 1.266 |  |  |  | 0.835 | 1.393 |
| Equation 3 | 0.372 | 0.023 | -0.140 | 0.319* | 0.128* | 1.312 | 0.685 |  |  | 0.831 | 1.407 |
| Equation 4 | 2.408 | 0.030 | -0.159 | 0.328* | 0.122* | 1.456 | 0.521 | 0.448 |  | 0.828 | 1.421 |
| Equation 5 | 5.391 | 0.038 | -0.156* | 0.332 * | 0.086 | 1.430 | 1.022 | 1.032 | -1.628* | 0.854 | 1.308 |
| Equation 6 | 4.997 | 0.048 | -0.159* | 0.336* | 0.096 |  |  | 1.057 | -1.530* | 0.856 | 1.297 |

^*^Denotes statistical significance (*P*<0.05)

Acronym: SEE, standard error of estimate
