# Supplementary material for: Development and cross-validation of prediction equations for body composition in adult cancer survivors from the Korean National Health and Nutrition Examination Survey (KNHANES)
Source: PLoS One. 2024 Oct 4;19(10):e0309061. doi: 10.1371/journal.pone.0309061 (PMC11451997; doi:10.1371/journal.pone.0309061)
Supplement: S8 Table — (DOCX) [file pone.0309061.s013.docx]

**Supplementary Table 8**. Anthropometric prediction equations for appendicular lean mass in the community-dwelling cancer survivors with obesity (body mass index≥25.0 kg/m^2^) derived the Korea National Health and Nutrition Examination Survey (2008-2011)

| Appendicular  lean mass |  |  |  |  |  |  |  |  |  |  |  |
| --- | --- | --- | --- | --- | --- | --- | --- | --- | --- | --- | --- |
|  | **Intercept** | **Age (years)** | **Height (cm)** | **Weight (kg)** | **Waist circumference (cm)** | **Creatinine**  **(mg/dL)** | **Smoking** | **Alcohol consumption** | **Physically inactive** | $\boldsymbol{R}^{\boldsymbol{2}}$ | **SEE** |
| Total(n=48) |  |  |  |  |  |  |  |  |  |  |  |
| Equation 1 | -46.489* | 0.045* | 0.345* | 0.114* | -0.003 |  |  |  |  | 0.856 | 1.324 |
| Equation 2 | -45.510* | 0.026 | 0.335* | 0.093 | 0.010 | 2.316 |  |  |  | 0.862 | 1.296 |
| Equation 3 | -43.781* | 0.026 | 0.328* | 0.104 | -0.002 | 1.903 | 0.702 |  |  | 0.863 | 1.292 |
| Equation 4 | -44.113* | 0.025 | 0.331* | 0.103 | -0.001 | 1.877 | 0.723 | -0.071 |  | 0.860 | 1.307 |
| Equation 5 | -44.627* | 0.021 | 0.330* | 0.103 | 0.004 | 2.026 | 0.819 | -0.242 | 0.429 | 0.858 | 1.314 |
| Equation 6 | -46.526* | 0.045 | 0.343* | 0.115 | -0.002 |  |  | -0.011 | 0.126 | 0.849 | 1.355 |
| Men(n=10) |  |  |  |  |  |  |  |  |  |  |  |
| Equation 1 | -13.155 | -0.040 | 0.287 | 0.082 | -0.165 |  |  |  |  | 0.591 | 1.537 |
| Equation 2 | -9.557 | -0.048 | 0.255 | 0.126 | -0.162 | -1.204 |  |  |  | 0.492 | 1.712 |
| Equation 3 | -18.716 | -0.094 | 0.328 | 0.143 | -0.135 | -5.243 | 1.242 |  |  | 0.383 | 1.886 |
| Equation 4 | -18.716 | -0.094 | 0.328 | 0.143 | -0.135 | -5.243 | 1.242 | 0.000 |  | 0.383 | 1.886 |
| Equation 5 | -43.538 | -0.130 | 0.563 | -0.099 | -0.157 | -0.613 | 2.694 | 0.000 | 3.091 | 0.663 | 1.394 |
| Equation 6 | -6.718 | -0.062 | 0.242 | 0.120 | -0.179 |  |  | 0.000 | 1.441 | 0.613 | 1.493 |
| Women(n=38) |  |  |  |  |  |  |  |  |  |  |  |
| Equation 1 | -28.670* | 0.021 | 0.222* | 0.152* | -0.007 |  |  |  |  | 0.865 | 0.881 |
| Equation 2 | -28.386* | 0.021 | 0.221* | 0.154* | -0.008 | -0.176 |  |  |  | 0.861 | 0.894 |
| Equation 3 | -31.777* | 0.024 | 0.236* | 0.138* | 0.015 | -0.256 | -1.207 |  |  | 0.869 | 0.870 |
| Equation 4 | -32.446* | 0.021 | 0.243* | 0.135* | 0.017 | -0.304 | -1.153 | -0.147 |  | 0.865 | 0.883 |
| Equation 5 | -33.827* | 0.018 | 0.241* | 0.134* | 0.034 | -0.292 | -1.385 | -0.418 | 0.754 | 0.874 | 0.851 |
| Equation 6 | -31.110* | 0.012 | 0.234* | 0.145* | 0.010 |  |  | -0.527 | 0.615 | 0.868 | 0.873 |

^*^Denotes statistical significance (*P*<0.05)

Acronym: SEE, standard error of estimate
