# Supplementary material for: Development and cross-validation of prediction equations for body composition in adult cancer survivors from the Korean National Health and Nutrition Examination Survey (KNHANES)
Source: PLoS One. 2024 Oct 4;19(10):e0309061. doi: 10.1371/journal.pone.0309061 (PMC11451997; doi:10.1371/journal.pone.0309061)
Supplement: S9 Table — (DOCX) [file pone.0309061.s014.docx]

**Supplementary Table 9**. Anthropometric prediction equations for body fat mass in the community-dwelling cancer survivors without obesity (body mass index<25.0 kg/m^2^) derived the Korea National Health and Nutrition Examination Survey (2008-2011)

| Body fat mass |  |  |  |  |  |  |  |  |  |  |  |
| --- | --- | --- | --- | --- | --- | --- | --- | --- | --- | --- | --- |
|  | **Intercept** | **Age (years)** | **Height (cm)** | **Weight (kg)** | **Waist circumference (cm)** | **Creatinine**  **(mg/dL)** | **Smoking** | **Alcohol consumption** | **Physically inactive** | $\boldsymbol{R}^{\boldsymbol{2}}$ | **SEE** |
| Total (n=107) |  |  |  |  |  |  |  |  |  |  |  |
| Equation 1 | -51.342* | 0.136 * | 0.383* | 0.544* | -0.153 |  |  |  |  | 0.780 | 3.261 |
| Equation 2 | -46.033* | 0.123* | 0.349* | 0.605* | -0.226* | 3.910* |  |  |  | 0.807 | 3.053 |
| Equation 3 | -40.745* | 0.106* | 0.318* | 0.611* | -0.219* | 3.043* | 2.270* |  |  | 0.820 | 2.945 |
| Equation 4 | -42.497* | 0.123* | 0.323* | 0.582* | -0.212* | 2.713* | 1.972* | 1.633* |  | 0.827 | 2.891 |
| Equation 5 | -42.504* | 0.123* | 0.323* | 0.582* | -0.212* | 2.716* | 1.968* | 1.632* | -0.022* | 0.825 | 2.906 |
| Equation 6 | -52.019* | 0.157* | 0.380* | 0.510* | -0.152 |  |  | 2.492* | -0.199 | 0.796 | 3.135 |
| Men(n=39) |  |  |  |  |  |  |  |  |  |  |  |
| Equation 1 | -4.110 | 0.098* | 0.088 | 0.779* | -0.250* |  |  |  |  | 0.860 | 2.040 |
| Equation 2 | -3.445 | 0.095* | 0.085 | 0.760* | -0.230* | -0.427 |  |  |  | 0.857 | 2.064 |
| Equation 3 | -2.864 | 0.095* | 0.076 | 0.784* | -0.243* | -0.446 | 0.750 |  |  | 0.857 | 2.058 |
| Equation 4 | -4.931 | 0.111* | 0.079 | 0.815* | -0.281* | -0.077 | 0.549 | 1.718 |  | 0.857 | 2.059 |
| Equation 5 | -1.038 | 0.096* | 0.048 | 0.815* | -0.261* | -0.468 | 0.774 | 1.659 | 1.290 | 0.866 | 1.995 |
| Equation 6 | -3.325 | 0.106* | 0.063 | 0.816* | -0.279* |  |  | 2.348 | 1.101 | 0.869 | 1.972 |
| Women(n=68) |  |  |  |  |  |  |  |  |  |  |  |
| Equation 1 | 1.048 | 0.012 | 0.088 | 0.388* | -0.053 |  |  |  |  | 0.569 | 2.187 |
| Equation 2 | -7.649 | 0.011 | 0.119 | 0.358* | -0.037 | 6.138* |  |  |  | 0.603 | 2.098 |
| Equation 3 | -4.171 | 0.012 | 0.097 | 0.365* | -0.040 | 6.013* | -1.654 |  |  | 0.607 | 2.088 |
| Equation 4 | -4.550 | 0.014 | 0.099 | 0.363* | -0.039 | 5.974* | -1.631 | 0.075 |  | 0.601 | 2.105 |
| Equation 5 | -5.686 | 0.018 | 0.098 | 0.352* | -0.022 | 7.222* | -1.782 | 0.035 | -0.864 | 0.606 | 2.091 |
| Equation 6 | -1.130 | 0.021 | 0.099 | 0.372* | -0.045 |  |  | 0.426 | -0.170 | 0.558 | 2.214 |

^*^Denotes statistical significance (*P*<0.05)

Acronym: SEE, standard error of estimate
