# Supplementary material for: Development and cross-validation of prediction equations for body composition in adult cancer survivors from the Korean National Health and Nutrition Examination Survey (KNHANES)
Source: PLoS One. 2024 Oct 4;19(10):e0309061. doi: 10.1371/journal.pone.0309061 (PMC11451997; doi:10.1371/journal.pone.0309061)
Supplement: S10 Table — (DOCX) [file pone.0309061.s015.docx]

**Supplementary Table 10**. Anthropometric prediction equations for lean body mass in the community-dwelling cancer survivors without obesity (body mass index<25.0 kg/m^2^) derived the Korea National Health and Nutrition Examination Survey (2008-2011)

| Lean body mass |  |  |  |  |  |  |  |  |  |  |  |
| --- | --- | --- | --- | --- | --- | --- | --- | --- | --- | --- | --- |
|  | **Intercept** | **Age (years)** | **Height (cm)** | **Weight (kg)** | **Waist circumference (cm)** | **Creatinine**  **(mg/dL)** | **Smoking** | **Alcohol consumption** | **Physically inactive** | $\boldsymbol{R}^{\boldsymbol{2}}$ | **SEE** |
| Total (n=107) |  |  |  |  |  |  |  |  |  |  |  |
| Equation 1 | 53.208* | -0.135* | -0.415* | 0.450* | 0.131 |  |  |  |  | 0.469 | 3.501 |
| Equation 2 | 47.315* | -0.121* | -0.377* | 0.382* | 0.213* | -4.340* |  |  |  | 0.539 | 3.261 |
| Equation 3 | 40.793* | -0.100* | -0.338* | 0.374* | 0.203* | -3.271* | -2.800* |  |  | 0.583 | 3.100 |
| Equation 4 | 42.350* | -0.115* | -0.343* | 0.400* | 0.197* | -2.977* | -2.536* | -1.451 |  | 0.593 | 3.065 |
| Equation 5 | 42.322* | -0.114* | -0.343* | 0.400* | 0.197* | -2.964* | -2.555* | -1.455 | -0.087 | 0.589 | 3.080 |
| Equation 6 | 53.885* | -0.156* | -0.411* | 0.484* | 0.130 |  |  | -2.481* | 0.159 | 0.501 | 3.391 |
| Men(n=39) |  |  |  |  |  |  |  |  |  |  |  |
| Equation 1 | 2.090 | -0.080 | -0.100 | 0.230 | 0.210 |  |  |  |  | 0.596 | 2.345 |
| Equation 2 | 1.721 | -0.079 | -0.098 | 0.240 | 0.199 | 0.237 |  |  |  | 0.584 | 2.378 |
| Equation 3 | 0.685 | -0.078 | -0.083 | 0.197 | 0.221 | 0.272 | -1.335 |  |  | 0.608 | 2.308 |
| Equation 4 | 2.770 | -0.094 | -0.086 | 0.167 | 0.260* | -0.100 | -1.133 | -1.732 |  | 0.605 | 2.317 |
| Equation 5 | -2.145 | -0.075 | -0.047 | 0.167 | 0.233 | 0.394 | -1.417 | -1.658 | -1.629 | 0.639 | 2.214 |
| Equation 6 | 0.978 | -0.089 | -0.070 | 0.187 | 0.242* |  |  | -2.675 | -1.346 | 0.625 | 2.259 |
| Women(n=68) |  |  |  |  |  |  |  |  |  |  |  |
| Equation 1 | -1.878 | -0.010 | -0.105 | 0.602* | 0.038 |  |  |  |  | 0.726 | 2.288 |
| Equation 2 | 5.994 | -0.009 | -0.133 | 0.630* | 0.024 | -5.556* |  |  |  | 0.741 | 2.224 |
| Equation 3 | 2.649 | -0.010 | -0.111 | 0.623* | 0.027 | -5.436* | 1.591 |  |  | 0.743 | 2.218 |
| Equation 4 | 1.665 | -0.006 | -0.106 | 0.616* | 0.029 | -5.539* | 1.651 | 0.195 |  | 0.739 | 2.235 |
| Equation 5 | 2.662 | -0.010 | -0.105 | 0.626* | 0.014 | -6.635* | 1.784 | 0.231 | 0.759 | 0.740 | 2.231 |
| Equation 6 | -1.173 | -0.013 | -0.108 | 0.608* | 0.034 |  |  | -0.140 | 0.116 | 0.717 | 2.324 |

^*^Denotes statistical significance (*P*<0.05)

Acronym: SEE, standard error of estimate
