# Supplementary material for: Development and cross-validation of prediction equations for body composition in adult cancer survivors from the Korean National Health and Nutrition Examination Survey (KNHANES)
Source: PLoS One. 2024 Oct 4;19(10):e0309061. doi: 10.1371/journal.pone.0309061 (PMC11451997; doi:10.1371/journal.pone.0309061)
Supplement: S11 Table — (DOCX) [file pone.0309061.s016.docx]

**Supplementary Table 11**. Anthropometric prediction equations for trunk fat mass in the community-dwelling cancer survivors without obesity (body mass index<25.0 kg/m^2^) derived the Korea National Health and Nutrition Examination Survey (2008-2011)

| Trunk fat mass |  |  |  |  |  |  |  |  |  |  |  |
| --- | --- | --- | --- | --- | --- | --- | --- | --- | --- | --- | --- |
|  | **Intercept** | **Age (years)** | **Height (cm)** | **Weight (kg)** | **Waist circumference (cm)** | **Creatinine**  **(mg/dL)** | **Smoking** | **Alcohol consumption** | **Physically inactive** | $\boldsymbol{R}^{\boldsymbol{2}}$ | **SEE** |
| Total (n=107) |  |  |  |  |  |  |  |  |  |  |  |
| Equation 1 | 25.556* | -0.057* | -0.234* | 0.247* | 0.116* |  |  |  |  | 0.495 | 1.988 |
| Equation 2 | 22.864* | -0.050* | -0.216* | 0.216* | 0.154* | -1.983* |  |  |  | 0.537 | 1.905 |
| Equation 3 | 19.745* | -0.040* | -0.198* | 0.212* | 0.149* | -1.471* | -1.339* |  |  | 0.565 | 1.846 |
| Equation 4 | 20.280* | -0.045* | -0.200* | 0.221* | 0.147* | -1.370* | -1.248* | -0.498* |  | 0.565 | 1.845 |
| Equation 5 | 20.263* | -0.045* | -0.199* | 0.221* | 0.147* | -1.363* | -1.260* | -0.500 | -0.052 | 0.561 | 1.854 |
| Equation 6 | 25.826* | -0.065* | -0.232* | 0.260* | 0.116* |  |  | -0.991* | 0.072 | 0.505 | 1.968 |
| Men(n=39) |  |  |  |  |  |  |  |  |  |  |  |
| Equation 1 | -0.563 | -0.035 | -0.063 | 0.115 | 0.157* |  |  |  |  | 0.521 | 1.651 |
| Equation 2 | -1.102 | -0.033 | -0.060 | 0.130 | 0.141 | 0.346 |  |  |  | 0.509 | 1.671 |
| Equation 3 | -1.685 | -0.033 | -0.052 | 0.106 | 0.154 | 0.366 | -0.752 |  |  | 0.522 | 1.649 |
| Equation 4 | -0.924 | -0.038 | -0.053 | 0.095 | 0.168 | 0.230 | -0.678 | -0.632 |  | 0.510 | 1.671 |
| Equation 5 | -4.058 | -0.026 | -0.028 | 0.095 | 0.151 | 0.545 | -0.859 | -0.585 | -1.039 | 0.539 | 1.619 |
| Equation 6 | -1.462 | -0.038 | -0.045 | 0.092 | 0.173* |  |  | -1.363 | -0.826 | 0.533 | 1.630 |
| Women(n=68) |  |  |  |  |  |  |  |  |  |  |  |
| Equation 1 | 2.377 | -0.001* | -0.110* | 0.328* | 0.079 |  |  |  |  | 0.666 | 1.564 |
| Equation 2 | 6.922 | -0.001 | -0.127* | 0.343* | 0.071 | -3.208 |  |  |  | 0.678 | 1.536 |
| Equation 3 | 5.458 | -0.001 | -0.117* | 0.340 * | 0.073 | -3.155 | 0.696 |  |  | 0.675 | 1.542 |
| Equation 4 | 3.903 | 0.004 | -0.108 | 0.330* | 0.076 | -3.318 | 0.792 | 0.309 |  | 0.672 | 1.550 |
| Equation 5 | 4.520 | 0.002 | -0.108* | 0.336* | 0.067 | -3.995* | 0.874 | 0.330 | 0.469 | 0.672 | 1.550 |
| Equation 6 | 1.733 | 0.00 | -0.106* | 0.324* | 0.079 |  |  | 0.122 | 0.089 | 0.656 | 1.588 |

^*^Denotes statistical significance (*P*<0.05)

Acronym: SEE, standard error of estimate
