# Supplementary material for: Development and cross-validation of prediction equations for body composition in adult cancer survivors from the Korean National Health and Nutrition Examination Survey (KNHANES)
Source: PLoS One. 2024 Oct 4;19(10):e0309061. doi: 10.1371/journal.pone.0309061 (PMC11451997; doi:10.1371/journal.pone.0309061)
Supplement: S12 Table — (DOCX) [file pone.0309061.s017.docx]

**Supplementary Table 12.** Anthropometric prediction equations for appendicular lean mass in the community-dwelling cancer survivors without obesity (body mass index<25.0 kg/m^2^) derived the Korea National Health and Nutrition Examination Survey (2008-2011)

| Appendicular  lean mass |  |  |  |  |  |  |  |  |  |  |  |
| --- | --- | --- | --- | --- | --- | --- | --- | --- | --- | --- | --- |
|  | **Intercept** | **Age (years)** | **Height (cm)** | **Weight (kg)** | **Waist circumference (cm)** | **Creatinine**  **(mg/dL)** | **Smoking** | **Alcohol consumption** | **Physically inactive** | $\boldsymbol{R}^{\boldsymbol{2}}$ | **SEE** |
| Total (n=107) |  |  |  |  |  |  |  |  |  |  |  |
| Equation 1 | -25.187* | 0.063* | 0.179* | 0.313* | -0.102* |  |  |  |  | 0.744 | 1.850 |
| Equation 2 | -22.319* | 0.056* | 0.161* | 0.346* | -0.141* | 2.112* |  |  |  | 0.772 | 1.745 |
| Equation 3 | -19.828* | 0.048* | 0.146* | 0.349* | -0.138* | 1.704* | 1.070* |  |  | 0.782 | 1.706 |
| Equation 4 | -20.764* | 0.057* | 0.149* | 0.333* | -0.134* | 1.527* | 0.910* | 0.873* |  | 0.788 | 1.681 |
| Equation 5 | -20.808* | 0.058* | 0.149* | 0.333* | -0.134* | 1.547* | 0.880 | 0.867 | -0.137 | 0.787 | 1.688 |
| Equation 6 | -25.532* | 0.075* | 0.177* | 0.295* | -0.101* |  |  | 1.305* | -0.206 | 0.760 | 1.790 |
| Men(n=39) |  |  |  |  |  |  |  |  |  |  |  |
| Equation 1 | 1.429 | 0.032 | 0.022 | 0.440* | -0.163* |  |  |  |  | 0.817 | 1.258 |
| Equation 2 | 1.541 | 0.031 | 0.021 | 0.437* | -0.160* | -0.072 |  |  |  | 0.812 | 1.277 |
| Equation 3 | 1.712 | 0.031 | 0.019 | 0.444* | -0.164* | -0.078 | 0.221 |  |  | 0.808 | 1.292 |
| Equation 4 | -1.253 | 0.054* | 0.022 | 0.488* | -0.219* | 0.452 | -0.067 | 2.464 |  | 0.832 | 1.205 |
| Equation 5 | 0.159 | 0.048 | 0.011 | 0.488* | -0.212* | 0.310 | 0.015 | 2.443* | 0.468 | 0.833 | 1.203 |
| Equation 6 | 0.848 | 0.044 | 0.008 | 0.472* | -0.194* |  |  | 2.288* | 0.508 | 0.842 | 1.170 |
| Women(n=68) |  |  |  |  |  |  |  |  |  |  |  |
| Equation 1 | 1.550 | 0.000 | 0.024 | 0.214* | -0.029 |  |  |  |  | 0.502 | 1.294 |
| Equation 2 | -3.594 | 0.000 | 0.043 | 0.196* | -0.020 | 3.631* |  |  |  | 0.541 | 1.242 |
| Equation 3 | -1.414 | 0.000 | 0.029 | 0.200* | -0.022 | 3.553* | -1.037 |  |  | 0.547 | 1.234 |
| Equation 4 | -0.908 | -0.001 | 0.026 | 0.204* | -0.023 | 3.605* | -1.068 | -0.101 |  | 0.540 | 1.243 |
| Equation 5 | -1.783 | 0.002 | 0.026 | 0.196* | -0.010 | 4.568* | -1.184 | -0.132 | -0.667 | 0.556 | 1.221 |
| Equation 6 | 0.961 | 0.004 | 0.027 | 0.208* | -0.024 |  |  | 0.120 | -0.225 | 0.489 | 1.311 |

^*^Denotes statistical significance (*P*<0.05)

Acronym: SEE, standard error of estimate
