# Supplementary material for: Development and cross-validation of prediction equations for body composition in adult cancer survivors from the Korean National Health and Nutrition Examination Survey (KNHANES)
Source: PLoS One. 2024 Oct 4;19(10):e0309061. doi: 10.1371/journal.pone.0309061 (PMC11451997; doi:10.1371/journal.pone.0309061)
Supplement: S13 Table — (DOCX) [file pone.0309061.s018.docx]

**Supplementary Table 13.** Concordance correlation coefficient for anthropometric prediction equations of body fat mass, lean body mass, trunk fat mass, and appendicular lean mass in the community-dwelling cancer survivors with obesity (body mass index≥25.0 kg/m^2^) derived the Korea National Health and Nutrition Examination Survey (2008-2011)

| CCC |  |  |  |  |
| --- | --- | --- | --- | --- |
|  | **Body fat mass** | **Lean body mass** | **Trunk fat mass** | **Appendicular**  **lean mass** |
| Total(n=48) |  |  |  |  |
| Equation 1 | 0.786 | 0.620 | 0.627 | 0.782 |
| Equation 2 | 0.791 | 0.654 | 0.642 | 0.792 |
| Equation 3 | 0.807 | 0.676 | 0.666 | 0.805 |
| Equation 4 | 0.803 | 0.661 | 0.652 | 0.805 |
| Equation 5 | 0.793 | 0.648 | 0.621 | 0.803 |
| Equation 6 | 0.781 | 0.609 | 0.608 | 0.781 |
| Men(n=9) |  |  |  |  |
| Equation 1 | 0.556 | 0.647 | 0.747 | 0.601 |
| Equation 2 | 0.647 | 0.611 | 0.778 | 0.705 |
| Equation 3 | 0.662 | 0.442 | 0.124 | 0.759 |
| Equation 4 | 0.662 | 0.442 | 0.124 | 0.759 |
| Equation 5 | 0.520 | 0.417 | 0.269 | 0.455 |
| Equation 6 | 0.567 | 0.639 | 0.725 | 0.599 |
| Women(n=39) |  |  |  |  |
| Equation 1 | 0.601 | 0.735 | 0.745 | 0.558 |
| Equation 2 | 0.578 | 0.727 | 0.747 | 0.559 |
| Equation 3 | 0.564 | 0.722 | 0.740 | 0.514 |
| Equation 4 | 0.562 | 0.699 | 0.726 | 0.519 |
| Equation 5 | 0.540 | 0.694 | 0.696 | 0.529 |
| Equation 6 | 0.573 | 0.697 | 0.699 | 0.572 |

Acronym: CCC, concordance correlation coefficient
