# Supplementary material for: Development and cross-validation of prediction equations for body composition in adult cancer survivors from the Korean National Health and Nutrition Examination Survey (KNHANES)
Source: PLoS One. 2024 Oct 4;19(10):e0309061. doi: 10.1371/journal.pone.0309061 (PMC11451997; doi:10.1371/journal.pone.0309061)
Supplement: S14 Table — (DOCX) [file pone.0309061.s019.docx]

**Supplementary Table 14.** Concordance correlation coefficient for anthropometric prediction equations of body fat mass, lean body mass, trunk fat mass, and appendicular lean mass in the community-dwelling cancer survivors without obesity (body mass index<25.0 kg/m^2^) derived the Korea National Health and Nutrition Examination Survey (2008-2011)

| CCC |  |  |  |  |
| --- | --- | --- | --- | --- |
|  | **Body fat mass** | **Lean body mass** | **Trunk fat mass** | **Appendicular**  **lean mass** |
| Total (n=107) |  |  |  |  |
| Equation 1 | 0.932 | 0.731 | 0.702 | 0.908 |
| Equation 2 | 0.939 | 0.764 | 0.723 | 0.917 |
| Equation 3 | 0.937 | 0.753 | 0.719 | 0.914 |
| Equation 4 | 0.932 | 0.750 | 0.721 | 0.911 |
| Equation 5 | 0.933 | 0.749 | 0.721 | 0.911 |
| Equation 6 | 0.923 | 0.722 | 0.705 | 0.901 |
| Men(n=42) |  |  |  |  |
| Equation 1 | 0.841 | 0.586 | 0.583 | 0.811 |
| Equation 2 | 0.836 | 0.582 | 0.575 | 0.810 |
| Equation 3 | 0.839 | 0.581 | 0.576 | 0.811 |
| Equation 4 | 0.831 | 0.575 | 0.571 | 0.781 |
| Equation 5 | 0.811 | 0.531 | 0.543 | 0.771 |
| Equation 6 | 0.810 | 0.536 | 0.547 | 0.768 |
| Women(n=65) |  |  |  |  |
| Equation 1 | 0.788 | 0.804 | 0.781 | 0.777 |
| Equation 2 | 0.799 | 0.812 | 0.778 | 0.777 |
| Equation 3 | 0.776 | 0.802 | 0.776 | 0.765 |
| Equation 4 | 0.776 | 0.801 | 0.770 | 0.766 |
| Equation 5 | 0.773 | 0.800 | 0.763 | 0.757 |
| Equation 6 | 0.785 | 0.804 | 0.781 | 0.775 |

Acronym: CCC, concordance correlation coefficient
