# Supplementary material for: Development and cross-validation of prediction equations for body composition in adult cancer survivors from the Korean National Health and Nutrition Examination Survey (KNHANES)
Source: PLoS One. 2024 Oct 4;19(10):e0309061. doi: 10.1371/journal.pone.0309061 (PMC11451997; doi:10.1371/journal.pone.0309061)
Supplement: S15 Table — (DOCX) [file pone.0309061.s020.docx]

**Supplementary Table 15.** Comparison of performance to prediction equations developed in the general population for body fat mass in the cross-validation set

|  | Difference  (DXA-equation) | SD | CCC | R^2^ | SEE |
| --- | --- | --- | --- | --- | --- |
| Park et al., (2024)^1^ |  |  |  |  |  |
| Men | -0.63 | 0.42 | 0.855 | 0.796 | 2.914 |
| Women | 0.03 | 0.23 | 0.840 | 0.722 | 2.287 |
| Lee et al.,(2017)^2^ |  |  |  |  |  |
| Men | 29.97 | 0.75 | 0.042 | 0.352 | 5.199 |
| Women | 11.56 | 0.39 | 0.190 | 0.512 | 3.029 |
| Lee et al.,(2021)^3^ |  |  |  |  |  |
| Men | 31.28 | 0.69 | 0.035 | 0.414 | 4.945 |
| Women | 14.46 | 0.34 | 0.128 | 0.550 | 2.910 |

NOTE: *Difference (DXA*-equation) refers to the actual body fat mass value in the cross-validation set minus the estimated value from the equation.

^1^ equation 1 (intercept, age, height, weight, waist circumference) from this study.

^2^ equation 2 (intercept, age, height, weight, waist circumference) from Lee et al., (2017) in the British Journal of Nutrition.

^3^ equation 1 (intercept, age, height, weight, waist circumference) from Lee et al., (2021) in Nutrition Research and Practice.

Acronym: SEE - standard error of estimate, CCC - concordance correlation coefficient
