# Supplementary material for: Development and cross-validation of prediction equations for body composition in adult cancer survivors from the Korean National Health and Nutrition Examination Survey (KNHANES)
Source: PLoS One. 2024 Oct 4;19(10):e0309061. doi: 10.1371/journal.pone.0309061 (PMC11451997; doi:10.1371/journal.pone.0309061)
Supplement: S16 Table — (DOCX) [file pone.0309061.s021.docx]

**Supplementary Table 16.** Comparison of performance to prediction equations developed in the general population for lean body mass in the cross-validation set

|  | Difference  (DXA-equation) | SD | CCC | R^2^ | SEE |
| --- | --- | --- | --- | --- | --- |
| Park et al., (2024)^1^ |  |  |  |  |  |
| Men | 0.66 | 0.44 | 0.774 | 0.634 | 3.070 |
| Women | -0.01 | 0.24 | 0.902 | 0.824 | 2.449 |
| Lee et al.,(2017)^2^ |  |  |  |  |  |
| Men | -33.97 | 0.75 | 0.018 | 0.175 | 4.607 |
| Women | -15.46 | 0.38 | 0.119 | 0.574 | 3.806 |
| Lee et al.,(2021)^3^ |  |  |  |  |  |
| Men | -36.45 | 0.71 | 0.021 | 0.269 | 4.336 |
| Women | -18.95 | 0.34 | 0.097 | 0.663 | 3.388 |

NOTE: *Difference (DXA*-equation) refers to the actual body fat mass value in the cross-validation set minus the estimated value from the equation.

^1^ equation 1 (intercept, age, height, weight, waist circumference) from this study.

^2^ equation 2 (intercept, age, height, weight, waist circumference) from Lee et al., (2017) in the British Journal of Nutrition.

^3^ equation 1 (intercept, age, height, weight, waist circumference) from Lee et al., (2021) in Nutrition Research and Practice.

Acronym: SEE - standard error of estimate, CCC - concordance correlation coefficient
